# Development and acceptability of a decision aid for anxiety disorder considering discontinuation of benzodiazepine anxiolytic

**DOI:** 10.3389/fpsyt.2023.1083568

**Published:** 2023-05-12

**Authors:** Yumi Aoki, Yoshikazu Takaesu, Ken Inada, Hiroki Yamada, Tomohiko Murao, Toshiaki Kikuchi, Masahiro Takeshima, Masayuki Tani, Kazuo Mishima, Tempei Otsubo

**Affiliations:** ^1^Department of Psychiatric and Mental Health Nursing, Graduate School of Nursing, St. Luke’s International University, Tokyo, Japan; ^2^Department of Neuropsychiatry, Kyorin University School of Medicine, Tokyo, Japan; ^3^Department of Neuropsychiatry, Graduate School of Medicine, University of the Ryukyus, Okinawa, Japan; ^4^Department of Psychiatry, Kitasato University School of Medicine, Sagamihara, Kanagawa, Japan; ^5^Department of Psychiatry, Showa University Northern Yokohama Hospital, Yokohama, Kanagawa, Japan; ^6^Yutaka Clinic, Kanagawa, Japan; ^7^Department of Neuropsychiatry, Keio University School of Medicine, Tokyo, Japan; ^8^Department of Neuropsychiatry, Akita University Graduate School of Medicine, Akita, Japan; ^9^Department of Psychiatry, Oouchi Hospital, Tokyo, Japan; ^10^Department of Psychiatry, Tokyo Women’s Medical University Adachi Medical Center, Tokyo, Japan

**Keywords:** anxiolytic, anxiety disorder, benzodiazepine, decision aid, shared decision making

## Abstract

**Aim:**

We aimed to develop a decision aid (DA) for individuals with anxiety disorders who consider tapering benzodiazepine (BZD) anxiolytics, and if tapering, tapering BZD anxiolytics with or without cognitive behavioral therapy (CBT) for anxiety. We also assessed its acceptability among stakeholders.

**Methods:**

First, we conducted a literature review regarding anxiety disorders to determine treatment options. We cited the results of the systematic review and meta-analysis, which we conducted previously, to describe the related outcomes of two options: tapering BZD anxiolytics with CBT and tapering BZD anxiolytics without CBT. Second, we developed a DA prototype in accordance with the International Patient Decision Aid Standards. We carried out a mixed methods survey to assess the acceptability among stakeholders including those with anxiety disorders and healthcare providers.

**Results:**

Our DA provided information such as explanation of anxiety disorders, options of tapering or not tapering BZD anxiolytics (if tapering, the options of tapering BZD anxiolytics with or without CBT) for anxiety disorder, benefits and risks of each option, and a worksheet for value clarification. For patients (*n* = 21), the DA appeared to be acceptable language (86%), adequate information (81%), and well-balanced presentation (86%). The developed DA was also acceptable for healthcare providers (*n* = 10).

**Conclusion:**

We successfully created a DA for individuals with anxiety disorders who consider tapering BZD anxiolytics, which was acceptable for both patients and healthcare providers. Our DA was designed to assist patients and healthcare providers to involve decision-making about whether to taper BZD anxiolytics or not.

## 1. Introduction

Anxiety disorders are common mental disorders characterized by emotional and stress reactions to a threat or anticipation of future concern (1), leading to a significant effect on a person’s physical and social functioning. Previous research revealed that individuals with anxiety disorders are associated with significant impairment to personal life (2) and quality of life (3), suicidal ideation and suicide attempts (4), and high care costs (5). Therefore, continued improvement in the care of people with anxiety disorders is important.

Benzodiazepine (BZD) anxiolytics are one of the treatment choices that are frequently used worldwide for the acute phase of anxiety disorders. However, the long-term BZD anxiolytic use is not recommended because of its disadvantages, including dependence (6), decline in cognitive functions (7), hip fractures associated with falls (8, 9), and impaired driving ability (10). Consequently, most anxiety disorder guidelines recommend that BZD anxiolytics should be used for only a short period (11–15). Moreover, some guidelines do not recommend the use of BZD anxiolytics, even for short-term periods, except in critical situations (16, 17).

Despite the evidence-based recommendations described above, BZD anxiolytics are commonly used worldwide for anxiety disorders (18, 19). Therefore, the safe discontinuation or tapering of BZD anxiolytics for anxiety disorders is essential. Thus, the establishment of treatment strategy against long-term BZD use for anxiety disorders may be warranted in clinical settings.

To address this issue, the evidence that psychological therapy is effective in reducing symptoms for anxiety disorders should be considered (20). Particularly, cognitive behavioral therapy (CBT) is an effective psychological intervention for anxiety disorders (21, 22). Several current guidelines recommend CBT as a first-line therapy because of its effectiveness in improving anxiety symptoms and comparatively fewer risks than BZD anxiolytics (11, 12, 17). Several trials assessing strategies for BZD discontinuation, such as gradual tapering or adding CBT, have reported the effectiveness of adding CBT in the short term (23). On the other hand, CBT has certain disadvantages, such as the lack of a fast-acting effect, longer consultation time, and high cost (24). Therefore, individuals with anxiety disorders deliberating on further non-medication treatment might face the advantages and disadvantages of CBT.

Approaches of treatment decision-making have shifted from the so-called paternalistic approach, where doctors take initiative in the decision-making, to patient-centered communication. In this type of approach, strategies such as “shared decision making” (SDM) have been emphasized, which focus on a patient’s value-based discussion that involves a two-way communication between the patient and their clinician about the positive and negative aspects of each treatment option (25, 26).

In relation to the SDM process, decision aids (DAs) have recently gained attention as patient-centered communication tools that promote two-way conversation between patients and healthcare providers during specific medical or mental conditions that require further treatment planning (27). DAs are intended to support individuals participating in the decision-making process by aiding them to make well-informed, preference-based choices when choosing their treatment options (27). DAs provide related information regarding the available options and aid people to solidify their own preferences, which are associated with different characteristics of each option (27). DAs can promote a patient’s involvement and increase concordance between their choices, preferences, and values during the decision-making process (28).

Various DAs, most of which were for decision-making during treatment initiation, have been developed in many areas including the somatic and psychiatric fields (28). Moreover, we developed several DAs for decision-making about whether the treatment should be continued or discontinued such as DA for depression remission (29) and DA for insomnia remission (30). Ramos-García et al. developed a Spanish version of DA for patients with generalized anxiety disorder (31), based on their needs that patients with GAD preferred an active and collaborative role in decision-making (32). However, to our best knowledge, there is no Japanese version of a DA for patients with anxiety disorders who are receiving BZD anxiolytics and considering further pharmacology treatment.

The aim of this study was to develop a Japanese version of DA for patients with anxiety disorders who are considering whether to discontinue BZD anxiolytics as well as whether to taper them with CBT or without CBT, if discontinuing BZD. The stakeholder’s acceptability of the DA were also examined. We have translated the DA into English so that many more people can utilize it.

## 2. Methods

### 2.1. Study design and conceptual framework

**Figure 1:**
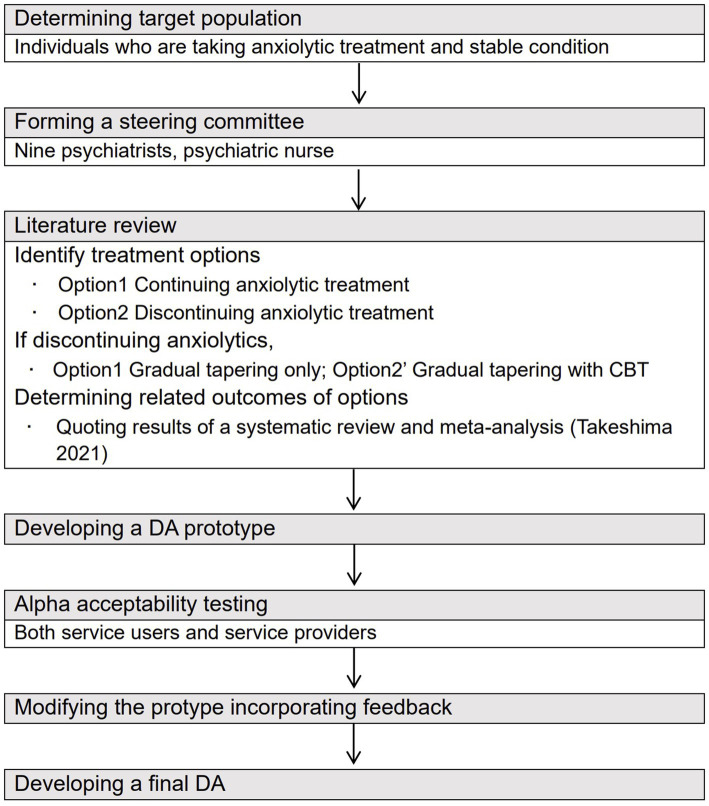
Process of developing a DA for those with anxiety disorder who consider tapering anxiolytics based on the approach of Coulter et al. (2013) (34).

The Ottawa Decision Support Framework (33) and International Patient Decision Aid Standards (IPDAS) were used to systematically develop the DA (34) (Figure 1). The IPDAS is one of the evidence-based frameworks that was established to standardize the development process and elements of DAs (35). The development process is as follows: (1) deciding the target people and assessing their decision-making needs, (2) establishing a steering committee made up by mental health professionals, (3) performing a literature review to decide the treatment options and related evidence-based outcomes, (4) creating a prototype of the DA, (5) assessing the acceptability of the prototype among stakeholders including patients and healthcare providers, (6) correcting the DA using the results of acceptability tests to create a final version of the DA, and (7) testing the developed DA for its effectiveness in clinical environment (35).

### 2.2. Determining the target population

The target people of the DA in this study was those who had been diagnosed with anxiety disorders, such as social anxiety disorder, generalized disorder, and panic disorder, and showed improvements in their symptoms and health conditions following treatment with BZD anxiolytics. Patients who were on medication but still experiencing symptoms were not targeted by the DA. The steering group expect that the DA would be useful in both primary care clinics and psychiatric outpatient clinics.

### 2.3. Assembling a steering committee members

The authors established a steering committee consisting of mental health professionals on anxiety disorders and DA methodology. The group was consisted of nine psychiatrists who regularly saw people with anxiety disorders and a psychiatric nurse who was familiar with SDM literature in the mental health field (36) and had experience creating DAs for mood disorders (29, 37), insomnia (30), and attention deficit hyperactivity disorder (38).

### 2.4. Literature review for exploring the related outcomes of each treatment option

The steering committee members examined the relevant published articles that explained anxiety disorders as a target disease and explored the advantages and disadvantages of the following treatment options: (1) continuing BZD anxiolytics, (2) tapering BZD anxiolytics, if tapering (3) gradually tapering BZD anxiolytics without CBT, and (4) gradually tapering BZD anxiolytics with CBT.

For the outcomes of the last two options, the committee referred to the results of a systematic review and meta-analysis that the authors had conducted and reported in detail elsewhere earlier (39). The meta-analysis indicated that CBT might be effective for stopping BZD anxiolytics, both in the short term (≤3 months) and long term (12 months) (39). Furthermore, references regarding the lifestyle changes that individuals with anxiety disorders can implement in daily life as self-management were also searched.

### 2.5. Developing the DA prototype

The committee members created a DA prototype according to the quality criteria of the IPDAS (33), citing the results of our literature review described above (39). DAs are basically of two types: one DA is for preparation for discussion with healthcare providers (designed to be used by patients at home) and the other DA is for conversation between patients and health care professionals to share decisions during clinical consultations (designed to encourage patients to be actively involved in conversations) (40). Our DA included both of those functions: preparation aid before consultation and conversation aid during consultation. For the preparation aid, the DA prototype provided queries to be selected by putting a check mark (worksheet for value clarification) and a box for any additional comments to be completed at home, which would be shared and discussed with their doctors during consultation. DAs should be understood by people who are unfamiliar with medical knowledge and therefore should be developed using eighth-grade level language (41). Considering this, the committee attempted to use simpler expressions. Moreover, in accordance with previously published evidence-based DAs, we described the outcome probabilities using pictograms, which showed how many people out of 100 would experience an event so that it could be easily understood by people with any literacy level (42).

### 2.6. Acceptability testing

We conducted acceptability testing of the DA prototype by surveying stakeholders. We adopted a mixed-methods survey.

Following a validated acceptability scoring measurement that assess the comprehensiveness of the DA in terms of its length, amount of information, balance of provided information, and ability to target decisions (43). This is the common DA development process that ensures the quality of the final version of the DA in accordance with stakeholder evaluation.

We recruited patients from the psychiatric outpatient departments of our university hospitals. Outpatients were approached if they fulfilled the following conditions: (i) aged ≥20 years, (ii) using BZD anxiolytics for at least 3 months, and (iii) showing improvements in their symptoms and health condition due to treatment with BZD anxiolytics. Furthermore, health care providers who regularly provided consultation to patients with anxiety disorders from the same department as those used by the outpatients were recruited. Approximately 20 individuals from each group were included in this study. The sample size was determined following the methods used in previous studies on DA development and acceptability testing (29, 30). Both the individuals with anxiety disorder and healthcare professionals were asked to read the DA prototype and participate in the survey. Finally, we modified and improved the DA prototype to create a final version using the results of acceptability testing.

## 3. Results

### 3.1. Components of the DA prototype

**Figure 2:**
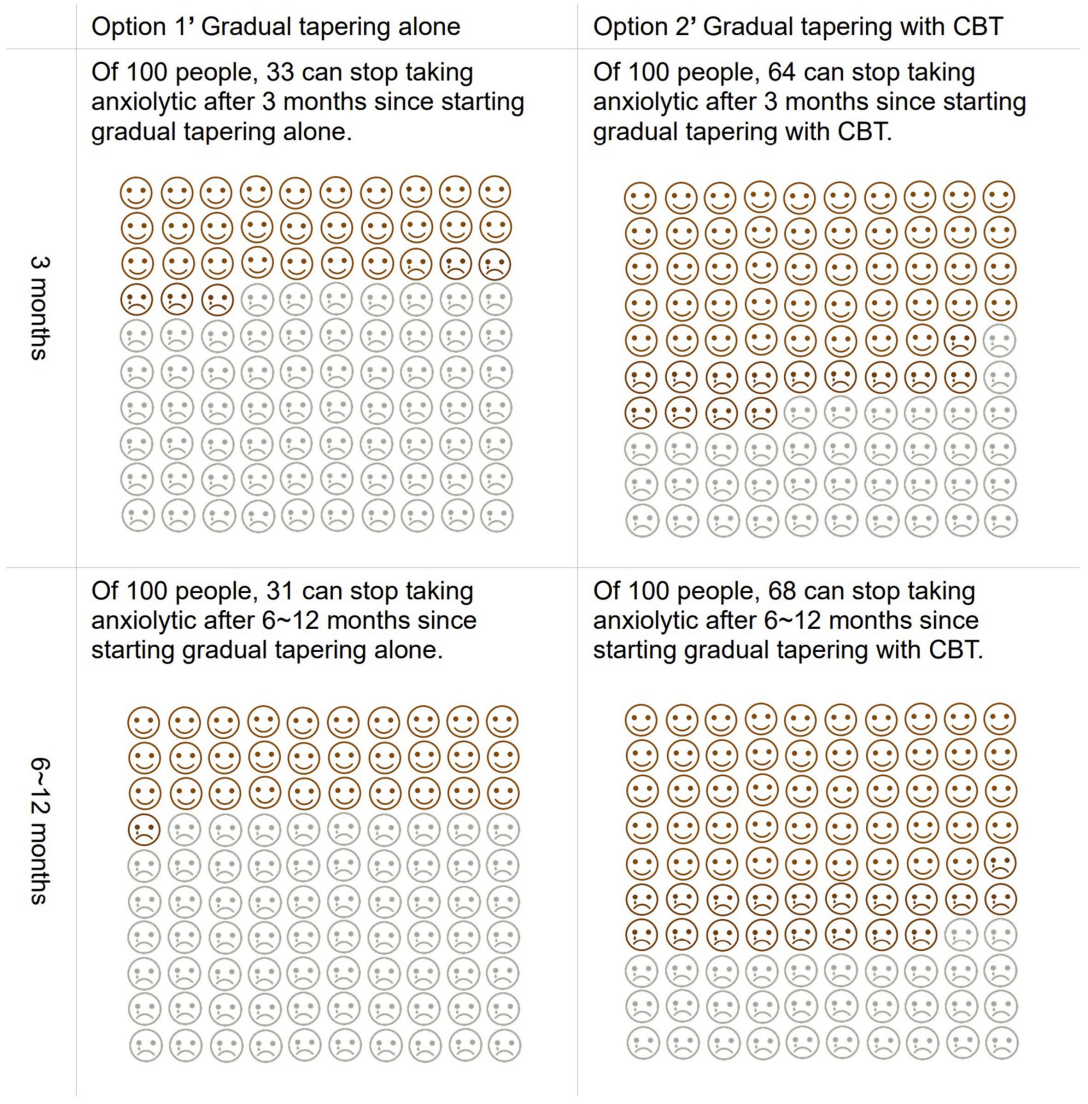
Pictorial diagrams as predicted consequences of tapering anxiolytics with and without CBT described in the DA.

Our DA prototype was a 32-page A5 booklet, which contained a description of the target people, instruction on how to use this tool, and an explanation of anxiety disorders. The prototype next provided the options of continuing (option 1) or tapering BZD anxiolytics (option 2), the advantages and disadvantages of each option, and a worksheet for value clarification. The booklet further prepared a box for those with anxiety disorders to put down any queries or comments to their clinicians, which could be asked in the next consultation on whether to continue or taper BZD anxiolytics. Additionally, for the tapering current anxiolytics option, the DA prototype showed additional options for gradually tapering BZD anxiolytics without CBT (option 1′) or with CBT (option 2′). For each option, the DA prototype recommended gradual tapering which involved reducing the dose by ≤25% over 4–8 weeks to prevent rebound anxiety, based on the current guidelines for BZD (15). Next, our DA described the advantages and disadvantages of these two options, along with a worksheet of value clarification for each option. The outcomes of each option were cited according to the outcomes of the meta-analysis that the authors had previously conducted, which found that gradual tapering with CBT was more effective than gradual tapering without CBT for success of stopping BZD anxiolytics both in the short-term (≤3 months) and long-term (12 months) (39). We described this evidence in the DA prototype using pictorial diagrams consisting of 100 faces, in which the number of colored faces meant the proportion of individuals who were predicted to experience the outcomes (Figure 2). Moreover, the DA prototype had a box for additional comments or queries to their clinicians, which could be asked in the next consultation on whether to taper BZD anxiolytics with CBT or without CBT. Supplementary material S1 showed the detailed information of the DA prototype.

### 3.2. Acceptability testing

#### 3.2.1. Patients

Twenty-one patients with anxiety disorders, such as general anxiety disorder (GAD) with sleep disorder (*n* = 6), GAD (*n* = 2), panic disorder (PD) (*n* = 2), PD with sleep disorder (*n* = 1), depression with GAD and sleep disorder (*n* = 1), depression with PD and sleep disorder (*n* = 1), PD with social anxiety disorder and sleep disorder (*n* = 1), and unknown (*n* = 7) participated in the DA acceptability testing. Ten patients (48%) were taking antidepressants as well as benzodiazepine anxiolytics, 4 (19%) were not, and 7 (33%) were unknown. Ten patients were taking hypnotics besides benzodiazepine anxiolytics, 4 (19%) were not taking them, and 7 (33%) were unknown whether to take them. Among the 21 patients, 14 (67%) have no CBT experience, while 7 (33%) were unknown. The mean age of the participants was 48.0 (±9.2) years, among which 14 (67%) were women, 5 (24%) were men, and 2 (10%) were unknown. Nine participants (43%) had a high school degree or lower level of education, 4 (19%) had vocational college level education, and 8 (38%) were university graduates.

Table 1 shows that the results of the patients’ feedback. The length of explanation or instruction was reported to be “just right” in 18 of 21 participants (86%). The amount of provided information was judged as “just right” in 17 of 21 participants (81%). The presentation of both options was rated as not biased but well balanced in 20 of 21 participants (95%). The DA was considered to be useful for decision-making about whether to taper anxiolytic drug or not in 17 of 20 patients (85%). A total of 14 of 20 patients (70%) thought that they could foresee their chance of successful stopping of current anxiolytics using the DA. Finally, 17 of 19 participants (89%) reported that the DA enabled easy decision making, while 18 of 21 participants (86%) thought that the DA had enough information to support to decide whether to continue or taper anxiolytics.

**Table 1:** Patient assessment on the way information is presented in each section of the prototype (*n* = 21).

	Mean	SD
About this booklet/Instructions on use	3.00	0.63
What is anxiety disorder?	3.29	0.56
Further treatment options	3.10	0.62
Comparing pros and cons of each option	3.10	0.77
Value clarification	3.19	0.81
Preparation for SDM	3.14	0.85
Appendices	3.43	0.51

SD, Standard Deviation. Rating system: four-point Likert scale from 1 to 4, 4 being excellent, 3 for good, 2 for fair, and 1 for poor.

In the comments from the participants, overall positive feedback on the DA prototype were observed. Some quotations are shown below.

*“I thought it was a good way to discuss and decide together.”* (Patient 8).

*“This is a good opportunity to fully think about anxiety disorder and my current treatment.”* (Patient 10).

*“I liked that it was explained in a way that made it easy for my family members who do not have a good knowledge about anxiety disorder to be able to read and understand it.”* (Patient 11).

*“This is good because I had felt that my doctors had not given me much detailed information about my treatment so far.”* (Patient 17).

*“I could understand my current condition. This booklet gave me an indication of what stage of treatment I was at.”* (Patient 19).

*“I thought it was good to be able to organize my thoughts and concerns in advance for the consultation.”* (Patient 20).

Furthermore, suggestions were provided to include additional explanations of some terms.

#### 3.2.2. Healthcare providers

Ten clinicians participated in the DA acceptability testing. The mean age of the clinicians was 37.3 (±10.1) years, and they included 2 (20%) women and 8 (80%) men.

The overall reaction of the DA prototype was preferable (Table 2). The comments from the clinicians contained several positive aspects of the DA prototype, including the concept of shared decision-making, visualization and friendly illustration, simple wording, and presentation of not biased either option.

**Table 2:** Healthcare providers’ perceptions of the DA prototype (*n* = 20).

	Mean	SD
It will be easy for me to use.	4.10	0.74
It is easy for me to understand.	4.30	0.48
It will be easy for me to experiment with using the strategy before making a final decision to adopt it (*n* = 19)	3.89	0.33
The results of using the strategy will be easy to see.	4.10	0.74
This strategy is better than how I usually go about helping patients decide about continuing or stopping anxiolytics.	4.20	0.79
This strategy is compatible with the way I think things should be done (*n* = 19)	4.33	0.71
The use of this strategy is a more cost-effective than my usual approach to helping patients decide about continuing or stopping anxiolytics	3.50	0.85
Compared with my usual approach, this strategy will result in my patients making more informed decisions.	4.70	0.48
Using this strategy will save me time.	3.80	1.14
This strategy is a reliable method of helping patients make decisions about continuing or stopping anxiolytics	4.40	0.52
Pieces or components of the strategy can be used by themselves.	3.70	0.67
This type of strategy is suitable for helping patients make value laden choices.	4.20	1.03
This strategy complements my usual approach.	3.70	1.16
Using this strategy does not involve making major changes to the way I usually do things.	3.90	0.57
There is a high probability that using this strategy may cause/result in more benefit than harm.	4.30	0.48

SD, Standard Deviation. Scored range from 1 = strongly disagree to 5 = strongly agree.

The examples of comments from clinicians are provided below.

*“I found the explanations with illustrations on how to taper off medication easy to understand.”* (Clinician 1).

*“I wanted to use it immediately in my clinic.”* (Clinician 4).

*“I did not know that I could make use of this kind of booklet before, so it’s a novelty.”* (Clinician 5).

*“It is nice that patients can gain basic knowledge about anxiety disorders and its treatment, which would help them to develop their own preferences and take the initiative in discontinuation decision-making.”* (Clinician 6).

*“I like that it describes alternative methods, such as breathing and relaxation techniques, along with medicines.”* (Clinician 9).

*“A detailed explanation of how this is used would be helpful.”* (Clinician 10).

### 3.3. Correcting the prototype incorporation stakeholder’s comments

The committee assembled and shared the results of the stakeholder’s acceptability test described above. We fully discussed and deliberated the results to utilize them to modify the DA prototype.

### 3.4. Developing the final DA

Our final DA was developed (Supplementary material S2) to ensure a high-quality decision support tool (Table 3). The final DA fulfilled all the IPDAS qualifying criteria (six of six), which were required for consideration as a DA (35), as well as all the IPDAS certification criteria (six of six), which judged the DA to contain a low risk of harmful bias (35). Moreover, the DA covered most IPDAS quality criteria (19 of 23), which added strength to the DA but whose lack did not mean a high risk of harmful bias (35). The status of the IPDAS criteria fulfilled by the final DA was considered higher than other Ottawa DAs that target other healthcare treatments or health screenings (44).

**Table 3:** International patient decision aid standards criteria met by current decision aid (30).

Item	1. Qualifying criteria	2. Certification criteria	3. Quality criteria
Information	Describes the health condition or problem for which decision is required ^a^	Shows the negative and positive features of options with equal detail ^a^	Describes the natural course of the health condition or problem if no action is taken ^a^
	Explicitly states decision that needs to be considered ^a^		Makes it possible to compare the positive and negative features of available options ^a^
	Describes the options available for the index decision ^a^		
	Describes positive features of each option ^a^		
	Describes negative features of each option ^a^		
Probabilities			Provides information about outcome probabilities associated with the options ^a^
			Specifies the defined group of patients for whom the outcome probabilities apply ^a^
			Specifies the event rates for outcome probabilities ^a^
			Allows the user to compare outcome probabilities across options using the same time period ^a^
			Allows the user to compare outcome probabilities across the same denominator ^a^
			Provides more than 1 way of viewing the probabilities (e.g., words, numbers, diagrams) ^a^
Values	Describes what it is like to experience consequence of the options ^a^		Asks patients to think about which positive and negative features of options matter most to them ^a^
Guidance			Provides a step-by-step way to make a decision ^a^
			Includes tools like worksheets or lists of questions to use when discussing options with a practitioner ^a^
Development			Development process included a needs assessment with clients or patients ^a^
			Development process included a needs assessment with health professionals ^a^
			Development process included review by clients/patients not involved in producing the decision support intervention ^a^
			Development process included review by professionals not involved in producing the decision support intervention ^a^
			Field tested with patients who were facing the decision ^b^
			Field tested with practitioners who counsel patients who face the decision ^b^
Evidence		Provides citations to the evidence selected ^a^	Describes how research evidence was selected or synthesized ^a^
		Provides a production or publication date ^a^	Describes the quality of the research evidence used ^a^
		Provides information about the update policy ^a^	
		Provides information about the levels of uncertainty around the event or outcome probabilities ^a^	
Disclosure		Provides information about the funding source used for development ^a^	Includes authors’/developers’ credentials or qualifications ^a^
Plain Language			Reports readability levels ^a^
Evaluation		Describes what the test is designed to measure ^b^	Evidence improved match between preferences of the informed patient and the option chosen ^b^
			Evidence patient decision aid helps patients improve their knowledge about options’ features ^b^

^a^Criteria met by the developed decision aid. ^b^Criteria to be met with effectiveness testing, not applicable for the current decision aid.

Additionally, the healthcare professionals who will be utilizing this DA will be required to be familiar with this tool. Therefore, the committee also created a DA manual for healthcare professionals that presented a detailed explanation of how to use the DA during decision-making in the clinical setting (Supplementary material S3).

## 4. Discussion

This is the first study to develop and assess the acceptability of a Japanese/English version of the DA for individuals with anxiety disorders for considering whether to continue BZD anxiolytics and whether CBT for anxiety should be added, if BZD is being discontinued.

The acceptability testing results suggested that the DA was well acceptable and favored by both patients and clinicians. This indicates that the DA was confirmed by stakeholders who were expected to use our DA. The strong point of the DA is that the committee systematically developed this tool using evidence-based criteria, in which both patients and clinicians, who were not involved in the development process, confirmed the DA. This implies that DA can be used in clinical settings. Ramos-García et al. also reported that their Spanish DA for patients with generalized anxiety disorder was easy to use, virtually appealing, and accepted by patients and clinical experts (31). These studies supported the suitability of DAs for anxiety-related disorders. Given that most people are highly motivated in contributing to the decision-making about their own treatment (32), these novel DAs could address the needs of patients with anxiety disorders.

The discontinuation of BZD anxiolytics has several advantages and disadvantages. The advantages include avoidance of adverse events, such as falls, drowsiness, and cognitive decline, whereas the disadvantages include worsening of anxiety and possible withdrawal symptoms. Thus, even if the patients desire to discontinue their medication, they may face conflicts between the advantages and disadvantages. Our DA might possibly reduce this conflict, since this tool successfully provides the evidenced-based characteristics of each option and asks the patients to clarify their own preferences. Using our DA with healthcare providers might also help patients to deliberate on further treatment courses with less conflict.

Several studies have been conducted to develop and assess psychosocial interventions for dealing with the risks of BZD use thus far (23). Heather et al. (45) reported that individuals with insomnia who received a letter warning about the harms of long-term use of BZD hypnotics showed larger reductions in BZD consumption than those who did not receive such a letter (23, 43). Thus, the presentation of not only the advantages but also the disadvantages of anxiolytic use to patients might lead to successful medication reduction. Our DA included both advantages and disadvantages of anxiolytics in a well-balanced manner. Moreover, our DA succeeded in supplying daily activities and relaxation techniques to reduce anxiety, which individuals with anxiety disorder could adopt in their everyday lives. In these regards, our DA contributes to the current literature, which suggests useful psychosocial interventions focusing on the prevention of the adverse aspects of long-term anxiolytic use. Furthermore, the uniqueness of our DA is that we have created a framework that allows patients to discuss and decide their options together with their clinicians, rather than unilaterally providing them with related information.

This study has some limitations. First, although our DA fulfilled most IPDAS quality criteria (35), some items should be covered in the future to improve the quality. Those items include field-testing and providing evidence of the intervention. To address this issue, the steering committee plans to conduct beta field-testing during the decision-making process of whether to discontinue BZD anxiolytics in a clinical setting. Second, there may be differences in the level of acceptance and appreciation among the patients who were shown their diagnosed disorder through the DA. Therefore, we plan to examine the differences between the diagnoses in beta field-testing. Third, patients with anxiety disorders often take antidepressant and BZD including some participants in this study. Therefore, there may be differences in the difficulties of discontinuing BZD if an antidepressant was also taken. We then plan to examine the differences between those on antidepressants and those who were not on antidepressants, in the beta field test. Forth, CBT for anxiety disorders include different elements and unique skills are required for each anxiety disorder. Our DA provided only non-specific general information of CBT for anxiety disorders, which is a limitation of this study. Additionally, the intervention effects of this DA need to be verified in a clinical setting.

## 5. Conclusion

This study described the development process and acceptability of a DA for the tapering BZD anxiolytics for anxiety disorders. The developed DA was acceptable to all stakeholders. The results could help in the treatment decisions of both individuals with anxiety disorder and their clinicians who are deliberating on the discontinuation of anxiolytic therapy.

## Data availability statement

The raw data supporting the conclusions of this article will be made available by the authors, without undue reservation.

## Ethics statement

The studies involving human participants were reviewed and approved by The Ethics Board of Kyorin University. The patients/participants provided their written informed consent to participate in this study.

## Author contributions

YA: study design, drafting and revising the DA prototype, data analysis and interpretation, revising the DA, and drafting the manuscript. KI, MTak, and TO: study design, revising the DA prototype, data collection, data analysis and interpretation, revising the DA, drafting, and editing the manuscript. HY, TM, TK, and MTan: study design, revising the DA prototype, data interpretation, revising the DA, and editing the manuscript. YT and KM: study design, revising the DA prototype, data collection and interpretation, revising the DA, editing the manuscript, and funding acquisition. All authors made substantial contributions to conception and design, acquisition of data, or analysis and interpretation of data, took part in drafting the article or revising it critically for important intellectual content, agreed to submit to the current journal, gave final approval of the version to be published, and agree to be accountable for all aspects of the work.

## Funding

This study was supported by research grants from the Ministry of Health, Labor and Welfare of Japan (19GC1012 and 21GC1016).

## Conflict of interest

YA received speaker’s honoraria from Sumitomo Pharma, Meiji Seika Pharma, Viatris Pharmaceuticals Japan. YT received a lecture sponsorship from Takeda Pharmaceutical, Sumitomo Pharma, Otsuka Pharmaceutical, Meiji Seika Pharma, Kyowa Pharmaceutical, Eisai, MSD, and Yoshitomi and re-search funding from Otsuka Pharmaceutical, Meiji Seika Pharma, MSD, and Eisai. KI has received personal fees/grant support from Eisai, Eli Lilly, Janssen, Meiji Seika Pharma, Mitsubishi Tanabe Pharma, Mochida, MSD, Novartis, Otsuka, Shionogi, Sumitomo Pharma, and Yoshitomiyakuhin in the last three years. HY received lecture fees from Takeda Pharmaceutical, Lundbeck Japan, Sumitomo Pharma, Otsuka Pharmaceutical, Meiji Seika Pharma, Janssen Pharma, Kyowa Pharmaceutical, Eisai, MSD, Yoshitomiyakuhin, Mochida Pharmaceutical and Viatris in the last three years. TM declares no interest of conflict. TK has received speaker’s honoraria from Takeda Pharmaceutical, Sumitomo Pharma, Viatris Pharmaceuticals Japan, MSD, Eisai, Ltd., and Yoshitomi Pharmaceutical. MTak has received speaker’s honoraria from Takeda Pharmaceutical, Otsuka Pharmaceutical, Daiichi Sankyo Company, Sumitomo Pharma, Meiji Seika Pharma, Viatris Pharmaceuticals Japan, MSD, Eisai, Ltd., and Yoshitomi Pharmaceutical, and research grants from Otsuka Pharmaceutical, Eisai, Shionogi and the Japanese Ministry of Health, Labour and Welfare (R3-21GC1016) outside the submitted work. MTan declares no interest of conflict. KM received speaker’s honoraria from EISAI Co., Ltd., Nobelpharma Co., Ltd., Takeda Pharmaceutical Co., Ltd., MSD Inc. and research grants from Eisai Co., Ltd., Sumitomo Pharma Co., Ltd., Takeda Pharmaceutical Co., Ltd., AMED (JP21dk0307103KM), the Japanese Ministry of Health, Labour and Welfare (19GC1012, 21GC0801). TO has received speaker’s honoraria from IQVIA, Takeda Pharmaceutical, Otsuka Pharmaceutical, Daiichi Sankyo Company, Sumitomo Pharma, Meiji Seika Pharma, Viatris Pharmaceuticals Japan, MSD, Eisai, Ltd., Kyowa Pharma, Lundbeck Japan, Lily, and Yoshitomi Pharmaceutical.

## Publisher’s note

All claims expressed in this article are solely those of the authors and do not necessarily represent those of their affiliated organizations, or those of the publisher, the editors and the reviewers. Any product that may be evaluated in this article, or claim that may be made by its manufacturer, is not guaranteed or endorsed by the publisher.
