# Supplementary material for: Development and acceptability of a decision aid for anxiety disorder considering discontinuation of benzodiazepine anxiolytic
Source: Front Psychiatry. 2023 May 12;14:1083568. doi: 10.3389/fpsyt.2023.1083568 (PMC10213963; doi:10.3389/fpsyt.2023.1083568)
Supplement: Supplementary file 1 [file Data_Sheet_1.PDF]

Supplement 1. Contents of the DA for anxiety disorder considering discontinuation of benzodiazepine anxiolytic.

**Contents**	**Pages**
About this booklet	
• Description of decision to be considered<br>• Explanation of target population<br>• Instructions for use of the booklet	1-3
What is anxiety disorders?	
• Objective information on anxiety disorders, such as panic disorder, social anxiety disorder, and general anxiety disorder	4
Step1 Further treatment options	
• Options provided: Continuing or discontinuing anxiolytics	6-8
Step2 Comparing each option	
• A table comparing each option (advantages and disadvantages)	9
Step3 Value clarification	
• A value clarification exercise with a 5-point Likert scale	10
Step 4 Preparation for shared decision making	
• Memo field to prepare for decision-making consultation	11
(If discontinuing medication) Step1 Further treatment options	
• Options provided: gradual tapering alone or gradual tapering with CBT<br>• Explanation of gradual tapering<br>• Explanation of CBT	12-14
(If discontinuing medication) Step2 Comparing each option	
• A table comparing each option (advantages and disadvantages)<br>• Pictorial diagrams comparing the consequences of each option	15,16
(If discontinuing medication) Step 3 Value clarification	
• A value clarification exercise with a 5-point Likert scale	17
(If discontinuing medication) Step 4 Preparation for shard decision making	
• Memo field to prepare for decision-making consultation	18
Appendix1 List of anxiolytics	
• Information on anxiolytics (characteristics of advantages and disadvantages)	19
Appendix2 About each anxiety disorder	
• General explanation of each anxiety disorder: panic disorder, social anxiety disorder, and general anxiety disorder	20-21
Appendix3 Lifestyle and behaviour changes to calm anxiety	
• Keeping an orderly life, deep breath, progressive muscle relaxation, imaging, positive coping skills for anxiety and stress	24,25
Appendix4 Frequently Asked Questions and Answers	
• Frequently asked questions and answers regarding anxiolytics	

DA, decision aid; CBT, Cognitive behavioural therapy
