# Supplementary material for: Development and acceptability of a decision aid for anxiety disorder considering discontinuation of benzodiazepine anxiolytic
Source: Front Psychiatry. 2023 May 12;14:1083568. doi: 10.3389/fpsyt.2023.1083568 (PMC10213963; doi:10.3389/fpsyt.2023.1083568)
Supplement: Supplementary file 2 [file Data_Sheet_2.PDF]

CONTINUING TAPERING STOPPINIG

A **decision aid** for anxiety disorders considering discontinuation of anxiolytics

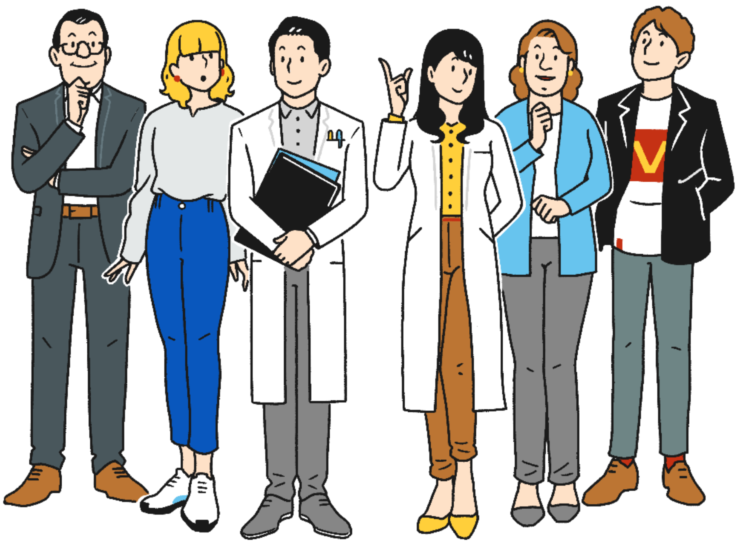

This decision aid is based on **the Guidelines for Psychotropic Drug Discontinuation Strategies**

Table of Contents

About this decision aid/How to use it? 1

What is anxiety disorder? 4

Treatment options for the future 6

Option1 Continuing anxiolytics 7

Option2 Reducing or stopping anxiolytics 8

Treatment options for reducing or stopping anxiolytics 12

Option1’ Reducing or stopping using gradual tapering method 13

Option2’ Reducing or stopping using gradual tapering and CBT 14

Appendix1 Common anxiolytics 19

Appendix2 About each anxiety disorder 20

Appendix3 Daily activities to reduce anxiety 23

Appendix4 Frequently asked questions and answers about anxiolytics 31

About this decision aid

This decision aid is designed to help individuals who are using anxiolytics for anxiety disorders, including panic disorder/agoraphobia, social anxiety disorder, and generalised anxiety disorder to determine further treatment with their healthcare providers. Anxiolytics are not recommended for long-term use because of their potential side-effects and dependence. Selective serotonin reuptake inhibitors (SSRIs) and serotonin noradrenaline reuptake inhibitors (SNRIs) are the main treatments for anxiety, and cognitive behavioural therapy (CBT) is becoming more commonly used. Some take anxiolytics in addition to SSRIs and SNRIs, whereas others take anxiolytics alone. Therefore, we developed this aid to help individuals and healthcare providers choose the most appropriate treatment method. This aid is designed to help individuals compare the pros and cons of each approach, to decide whether to continue, reduce, or stop anxiolytics, and to determine whether to undergo CBT when reducing or stopping anxiolytics.

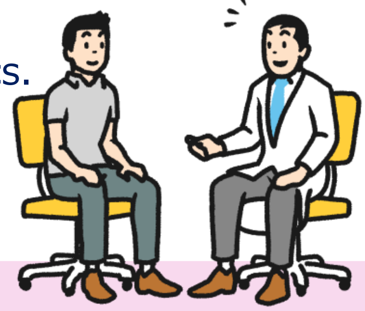

About this decision aid

【Those who are eligible for this aid】

・Individuals who are taking anxiolytics*, whose symptoms of anxiety have generally improved, and who are in good physical and mental condition.

・Although the main target group is individuals using anxiolytics alone, those taking psychotropics in addition to anxiolytics are also eligible.

【Those who are NOT eligible for this aid】

・Individuals whose symptoms of anxiety have not sufficiently improved.

・Individuals who are not in good physical or mental condition.

*Common anxiolytics covered in this aid are provided in Appendix1. Please check if you are taking any of these medicines.

If you are not sure if you are eligible for this aid, please consult your doctor.

How to use this decision aid?

This decision aid is meant for discussion with the healthcare provider and determination of the future treatment. You can take this aid home, read it carefully, and discuss it with their family members; therefore, the future treatment plan may be contemplated together.

This decision aid should be read carefully

Answers should be marked using a circle or the memo fields should be filled

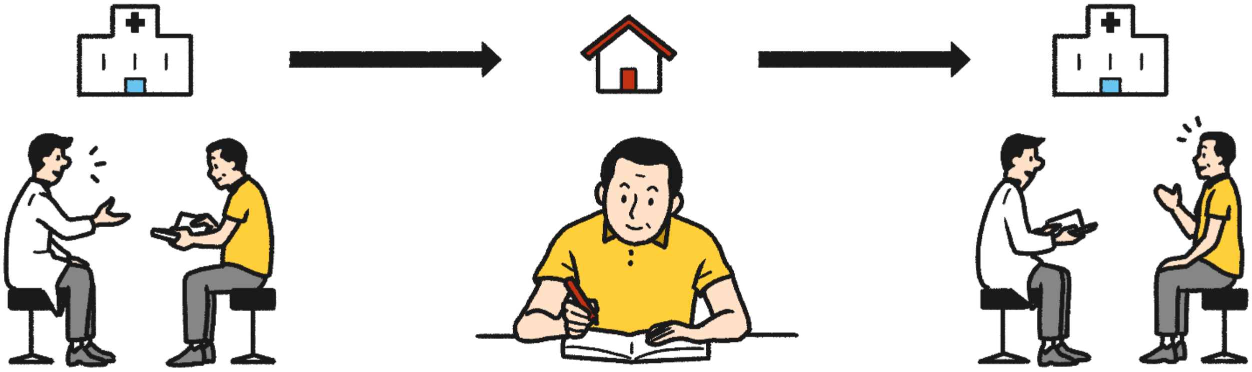

During consultation

- Your current condition will be explained
- Treatment options will be reviewed

During consultation

- The circled items and the contents of the memo should be discussed.
- Further treatment should be planned.

※If we can't decide at once, it should be brought back for further consideration.

What are anxiety disorders?

Typical anxiety disorders include panic/agoraphobia, social anxiety disorder and generalized anxiety disorder. The different types of anxiety disorder have different symptoms and mechanisms.

Panic disorder is an anxiety disorder characterised by sudden panic attacks, anticipatory anxiety that the anxiety will occur again, and agoraphobia, which causes avoidance of certain places and situations.

Social anxiety disorder is an anxiety disorder that causes intense anxiety and fear of social situations that may attract the attention of others.

General anxiety disorder is an anxiety disorder in which chronic uncontrollable worry makes it difficult to function in daily life.

Ask your doctor which anxiety disorder you have. The different anxiety disorders are listed in Appendix 2. Please refer to this list.

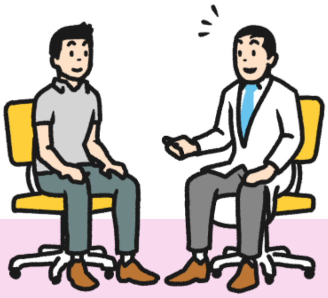

What is anxiety disorder?

If anxiety does not improve with your coping skills in daily routines, monotherapy with SSRIs or SNRIs is recommended. In addition, some may use anxiolytics (benzodiazepines). Anxiolytics should not be used for an extended period because of their side effects. Other common difficulties with taking anxiolytics include dependence and withdrawal symptoms when they are discontinued. Thus, particular attention should be paid when anxiolytics are used without SSRIs and SNRIs.

Once your anxiety has improved and your physical and mental condition during daytime has been stable, you may wish to consider whether to continue taking anxiolytics or to reduce their dose as a step toward discontinuing the medication.

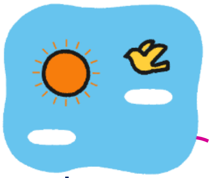

Next, we will look at further treatment options when anxiety has improved and the daytime physical and mental condition is stable. ☞

Further treatment options

Step 1 Each option should be understood

Option 1 Continue anxiolytics

What to do when you chose this option:

- Continuing anxiolytics ➡ page **7**
- Daily activities should be continued, which assist in reducing anxiety ➡ Appendix**3**
- Continuing SSRI/SNRI (if taking)

Option 2 Reducing or stopping anxiolytics

What to do when you chose this option:

- Reducing or stopping anxiolytics ➡ page **8**
- Daily activities should be continued, which assist in reducing anxiety ➡ Appendix**3**
- Continuing SSRI/SNRI (if taking)

Option 1 Continuing anxiolytics

Pros and cons when continuing anxiolytics are provided.

Pros

When continuing anxiolytics:

・Can maintain current stable condition where anxiety has been improved

・Can maintain current daily life and mental/physical stability

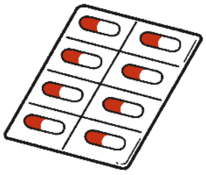

Cons

When continuing taking anxiolytics:

・Side effects (e.g. falls, drowsiness, cognitive decline, amnesia) and dependence may occur with anxiolytics.

・It may need to be careful with other medicines.

・It may affect pregnancy and breastfeeding.

Option2 Reducing or stopping anxiolytics

It is not recommended to take anxiolytics for a long period of time. When your anxiety symptoms have improved, you are not experiencing difficulties in daily life, and your physical and mental condition during daytime is stable, it is a good time to consider reducing or discontinuing the dose.

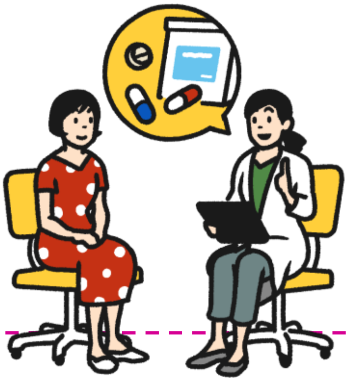

Next, the pros and cons of the options 'continuing anxiolytics' and 'reducing or stopping anxiolytics’ are compared.

Pros and Cons of Each Option

Step 2 Compare the pros and cons of each option

	Option1<br>**Continuing anxiolytics**<br>・Continuing anxiolytics<br>・Continuing daily activities for to reduce anxiety⇒Appendix3	Option2<br>**Reducing/stopping anxiolytics**<br>・Reducing/stopping anxiolytics<br>・Continuing daily activities to reduce anxiety⇒Appendix3
**Pros**	・May be useful for maintaining your current condition, in which anxiety has improved<br>・Not suffering from withdrawal symptoms※	・No more fear of not being able to stop anxiolytics<br>・No more side effects※※ and dependence
**Cons**	・Persistent anxiety about not being able to stop anxiolytics<br>・May suffer from side effects※※ and dependence	・Anxiety symptoms and mental/physical conditions may worsen<br>・Withdrawal symptoms※ may occur when the dose is reduced

※Withdrawal symptoms: nausea, sweating, dizziness, anxiety, gastrointestinal symptoms etc.

※※Side effects: risk of falls, drowsiness, possible cognitive decline, or amnesia etc.

What is important to you?

Step 3 Organise what is important

Below we have listed the main reasons for choosing each option. The importance of each of these to you should be analysed. The numbers that are applicable should be encircled with your importance.

Option 1. Reason for 'continuing anxiolytics'

	Not important					Important
Anxiety has improved	0	1	2	3	4	5
May be useful for maintaining your current condition	0	1	2	3	4	5
Others (please state freely)						
·	0	1	2	3	4	5
·	0	1	2	3	4	5

Option 2. Reason for 'reducing or stopping anxiolytics'

	Not important					Important
No more fear of not being able to stop anxiolytics	0	1	2	3	4	5
No more side effects and dependence	0	1	2	3	4	5
Others (please state freely)						
·	0	1	2	3	4	5
·	0	1	2	3	4	5

Preparing for a consultation discussion

Step 4 Preparing for the discussion with the doctor

Based on the thoughts and weightage, we will discuss option 1 'continue anxiolytics' and option 2 'reduce or stop anxiolytics' in the consultation.

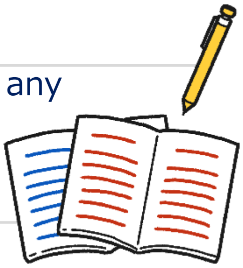

Please note down any current feelings and thoughts, including any questions or concerns.

If you would like to 'reduce or stop anxiolytics', kindly check the next page.☞

When reducing or stopping anxiolytics

Further treatment options

Step 1 Understand what each option is

Option 1′ Reducing or stopping using gradual tapering method

What to do when you choose this option?

- You should continue performing daily activities, which assist in reducing anxiety※
- The pills should be reduced or stopped using the gradual tapering method ➡ **page 13**

Option 2′ Reducing or stopping using gradual tapering and cognitive behavioural therapy (CBT)

What to do when you choose this option?

- You should continue performing daily activities, which assist in reducing anxiety※
- The pills should be reduced or stopped using the gradual tapering method ➡ **page 13**
- You should take CBT too ➡ **page 14**

※ Examples of daily activities to assist in reducing anxiety
➡ Appendix 3

Option 1'
Reducing or stopping using gradual tapering

When anxiolytics are stopped abruptly, anxiety may become more intense than it was before starting. This is called rebound anxiety. To prevent this, a gradual reduction in dosage is called a taper. This aims to reduce the dose gradually; therefore, you can eventually survive without the medication.

【How to reduce or taper medicines?】

- The dose should be gradually reduced by half a tablet over a period of 2–4 weeks.
- In the first few days, anxiety may be enhanced; however, this will gradually improve as the anxiety subsides.
- If there is rebound anxiety, you should go back one level.

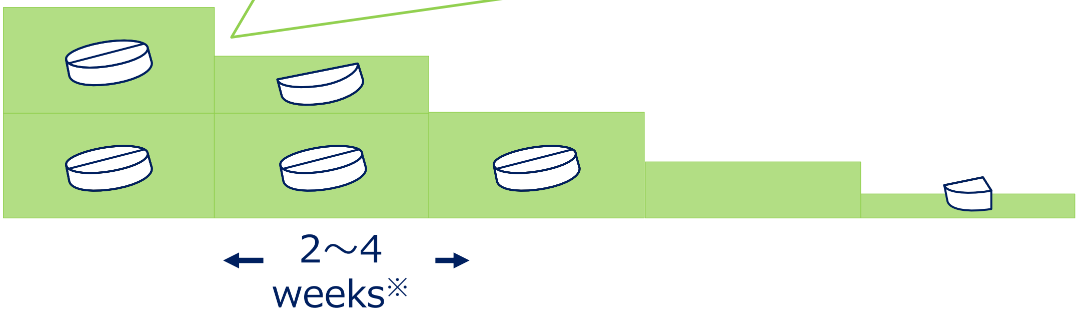

※The duration is a guide. It will be reduced slowly in consultation with the doctor.

After stopping, you may feel uncomfortable than before when the drug was administered; however, this is not a problem as long as it does not interfere with the daytime activities.

Option 2′
Reducing or stopping using gradual tapering and CBT

CBT is a treatment that aims to improve anxiety by improving the cognitions (thoughts) and lifestyle habits that interfere your life and by learning relaxation techniques.

It involves 4–6 sessions of 60 min once weekly with a trained professional (doctor, licensed psychologist, or nurse). For details of CBT for each anxiety disorder, see the website of Japanese Society of Anxiety and Related Disorders.

jpsad.jp/manual.php

Although it does not have the same immediate effect as anxiolytics, it is as effective as anxiolytics for anxiety disorders. It has also been reported that it is easier to reduce or stop medication when used in conjunction with the tapering method. Currently, however, there are only a limited number of facilities that offer this treatment. Panic disorder and social anxiety disorder may be covered by medical insurance.

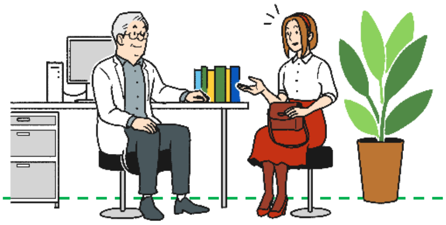

Now let us compare the pros and cons of the two options: reducing or stopping anxiolytics using tapering method or using a combination of tapering and CBT.☞

Pros and Cons of Each Option

Step 2 The pros and cons of each option should be compared

	**Option 1′**<br>**Reducing or stopping using gradual tapering**<br>• Reducing or stopping using gradual tapering<br>• Continue performing daily activities, which assist in reducing anxiety ⇒Appendix3	**Option 2′**<br>**Reducing or stopping using gradual tapering and CBT**<br>• Reducing or stopping using gradual tapering<br>• Taking CBT<br>• Continue performing daily activities, which assist in reducing anxiety ⇒Appendix3
**Pros**	・Can be performed under current doctor's care<br>・Can be conducted within the scope of insurance treatment<br>・Does not require time and effort besides medical treatment	Compare to 'option 1',<br>・Easier to reduce anxiolytics<br>・May be less likely to experience worsening of anxiety symptoms after reducing or stopping anxiolytics
**Cons**	Compare to 'option 2',<br>・May be difficult to reduce or discontinue anxiolytics<br>・May worsen anxiety symptoms	CBT：<br>・May need to change to another healthcare provider<br>・May involve additional costs, which may not be covered by insurance<br>・May require time and effort to realise the benefits

The consequences of each option

Step 3 We compare the consequences of each option

Here are our estimates of what percentage of people on anxiolytics would be able to stop if they chose each option. Each face represents one person and shows how many people out of 100 would stop anxiolytics.

	**Option 1'** **Gradual tapering alone**	**Option 2'** **Gradual tapering with CBT**
3 months	Of 100 people, 33 can stop taking anxiolytics after 3 months since starting gradual tapering alone. 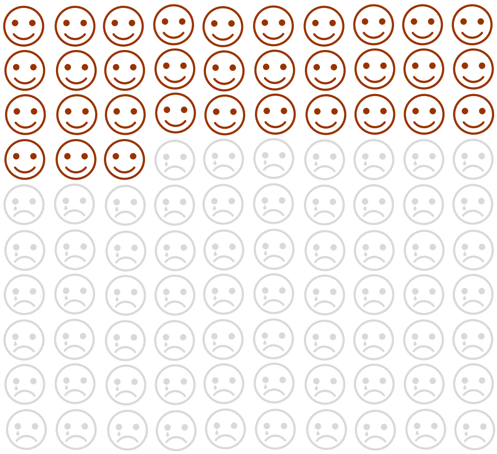	Of 100 people, 64 can stop taking anxiolytics after 3 months since starting gradual tapering with CBT. 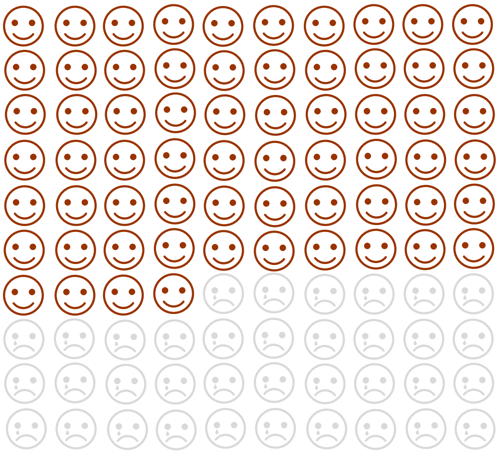
12 months	Of 100 people, 31 can stop taking anxiolytics after 12 months since starting gradual tapering alone. 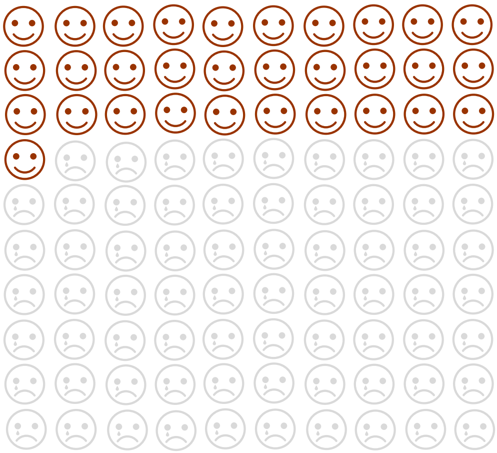	Of 100 people, 49 can stop taking anxiolytics after 12 months since starting gradual tapering with CBT. 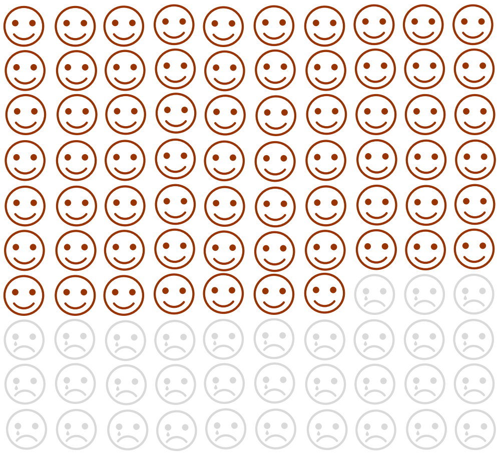

What is important to you?

Step 4 Organise what is important

Below we have listed the main reasons for choosing each option. The importance of each of these to you should be analysed. The numbers that are applicable should be encircled with your importance.

Option 1’ Reason for 'Gradual tapering alone'

	Not important					Important
• Can be performed under current doctor’s care	0	1	2	3	4	5
• Can be conducted within the scope of insurance treatment	0	1	2	3	4	5
Other (please state freely)						
•	0	1	2	3	4	5
•	0	1	2	3	4	5

Option 2’ Reason for 'Gradual tapering with CBT'

	Not important					Important
• Easier to reduce anxiolytics	0	1	2	3	4	5
• Less likely to feel anxiety	0	1	2	3	4	5
Other (please state freely)						
•	0	1	2	3	4	5
•	0	1	2	3	4	5

Preparing for a consultation discussion

Step 5 Preparing for the discussion with the doctor

Based on the thoughts and weightage, we will discuss **option1'** 'Gradual tapering alone' and **option2'** 'Gradual tapering with CBT' in the consultation.

Please note down any current feelings and thoughts, including any questions or concerns.

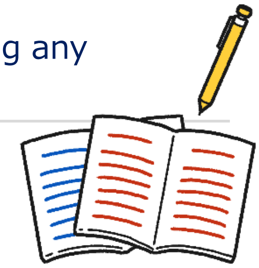

Appendix1 Common anxiolytics

Appendix2 About each anxiety disorder

Appendix3 Daily activities for reducing anxiety

Appendix4 Frequently asked questions and answers about sleeping pills

Appendix1 Common anxiolytics

The following table shows the most common anxiolytics.
The table should be used to check with the doctor about the characteristics of the medications you are taking.

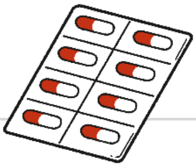

Short ↑ Duration of action ↓ Long

Duration of action	Generic name【Trade name】
Short-acting	Etizolam [Depas®]<br>Clothiazepam [Liese®]<br>Flutazolam [Choluminar®]
Intermediate acting	Lorazepam [Waipax®]<br>Alprazolam [Constan®/Solanax®]<br>Fludiazepam [Ellispan®]<br>Bromazepam [Rexotan®/Selanin®]<br>Diazepam [Selsyn®/Horizon®]
Long-acting	Cloxazolam [Cepazone®]<br>Chlordiazepoxide [Kontol®]<br>Medazepam [Resmit®]<br>Furtoprazepam [Restus®]
Ultra-long acting	Ethyl roflazepate [Mayrax®]<br>Mexazolam [Melex®]<br>Oxazolam [Serenal®]

Pros Reducing anxiety, relatively low cost
[Short-acting drugs] are less likely to exhibit accumulation.
[Long-acting drugs] can reduce the frequency of use.

Cons Gradual loss of efficacy, possible side effects (e.g., falls, drowsiness, cognitive decline, forgetfulness, withdrawal symptoms (e.g., nausea, vomiting, sweating, dizziness, anxiety) when stopping, problems related to dependence
[Short-acting drugs] more likely to cause withdrawal symptoms when interrupted after continuous use.
[Long-acting drugs] may accumulate in the body with repeated use; withdrawal symptoms are less noticeable.

Panic disorder／agoraphobia

What is panic disorder/agoraphobia?

Panic disorder is a mental disorder characterised by the experience of anxiety. Panic attacks refer to the occurrence of physical symptoms such as palpitations, breathing difficulties, sweating, dizziness, trembling and a feeling of suffocation, along with a fear of death or going insane, lasting for a limited duration. Panic attacks are characterised by sudden and recurrent onset. Panic disorder is complicated by “anticipatory anxiety”, which is a fear of another panic attack, and “agoraphobia”, which refers to the fear and avoidance of situations and places from which one cannot seek help or escape in the event of a panic attack. The prevalence of panic disorder is approximately 1.7%–3.3% in Japan and is thought to be two to three times higher in women. In women, panic disorder is more common among individuals in their 20s, 30s, and 50s–60s. In men, the prevalence of panic disorder is thought to decrease after their 30s.

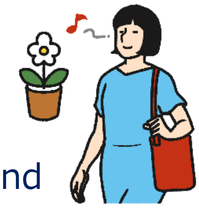

Treatment of panic disorder/agoraphobia

Treatment options for panic disorder include medication and psychotherapy, including cognitive-behavioural therapy (CBT). Regarding medication, SSRIs are currently recommended as a first-line treatment, but because the onset of effect takes time (2–6 weeks), benzodiazepines, which are fast-acting anxiolytics, can be used in the short term. However, benzodiazepines must be used with caution because of problems of dependence and abuse, and long-term use is not recommended.

Non-pharmacological therapies include psychoeducation and CBT, which can be effective for those who do not respond to medication. Other lifestyle modifications are also important, such as avoiding stress and stimulants that make panic attacks more likely.

Social anxiety disorder

What is social anxiety disorder?

Social anxiety disorder (SAD) is a condition in which an individual feels intense anxiety and fear regarding social situations that may attract attention from others. Fear of negative feedback (e.g., thoughts such as “I will be embarrassed or rejected by others because of my behaviour or insecurity”) can lead to avoidance of social situations such as meetings and presentations, which can interfere with daily life and social interaction. For a diagnosis of SAD, these symptoms must last for at least 6 months. The age of onset is early, peaking in the teenage years, and symptoms are usually long-lasting.

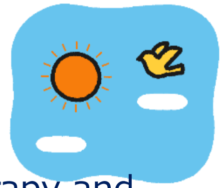

Treatment of social anxiety disorder

Treatment of SAD typically consists of pharmacotherapy and psychotherapy, including CBT, which have similar short-term effects. In Japan, antidepressants and anxiolytics are commonly used as pharmacotherapy, with antidepressants being the first choice. Anxiolytics involve several difficulties, including dependence. Thus, short-term use is recommended only for people who have only just begun treatment or who have failed to respond to other treatments, and those who have severe symptoms and require rapid symptom relief.

General anxiety disorder

What is general anxiety disorder?

General anxiety disorder (GAD) is a condition characterised by chronic uncontrolled “worrying” (imagining things that may or may not happen and worrying with a strong sense of urgency). This disorder causes physical symptoms such as an inability to sleep well, muscle tension and stiffness, and difficulty concentrating, which can lead to serious social and occupational functioning problems, development of other psychiatric disorders, and an increased risk of suicide. However, diagnosis of GAD tends to be delayed because the symptom of “worry” is often also exhibited in depression or other anxiety disorders.

Treatment of general anxiety disorder

The main treatments for GAD are medication and psychotherapy, including CBT. Antidepressants are considered to be the first-line drug treatment for GAD, particularly SSRIs and SNRIs. Other drugs, such as anxiolytics, are also used, but because of side effects and dependence, the use of anxiolytics is recommended only in the early stages of treatment (within 1 month) before the onset of antidepressant effects. Psychotherapy and pharmacotherapy are equally important treatment methods. The basic approach of psychotherapy focuses on acceptance and empathy, using a micro-psychotherapeutic approach. On this basis, CBT is recommended if necessary.

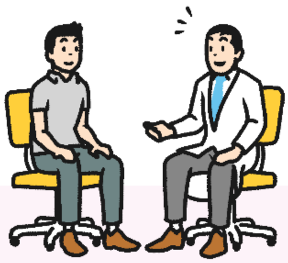

Appendix3 Daily activities to reduce anxiety

Anxiety and tension can be eased in everyday life.

1. The basics

1. Maintaining a regular rhythm of life, going to bed at a regular time, getting up at a regular time, and eating three meals a day.

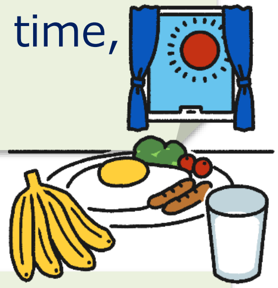

2. Avoiding drinking too much alcohol, smoking too many cigarettes, and consuming too many stimulants like caffeine.

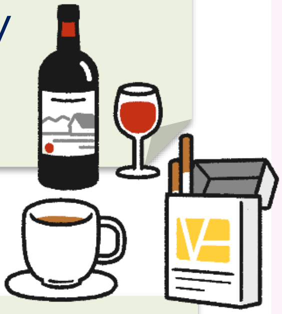

3. Being careful not to overexert yourself and performing a moderate amount of exercise.

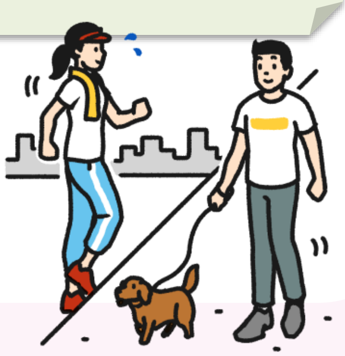

2. Relaxation techniques

The experience of often involves shallow breathing and high muscle tension. Relaxing the mind and body on a regular basis can also help to relieve anxiety. Some examples are presented below.

Breathing exercises

Inhale through the nose, then stop, and exhale through the mouth. You can adjust the amount of time yourself. It is recommended to perform these exercises two to three times a day. The three key points are as follows: (1) exhale long and slow, (2) perform abdominal breathing, and (3) when you exhale, imagine that you are exhaling anxiety, tension, and stress out of your body.

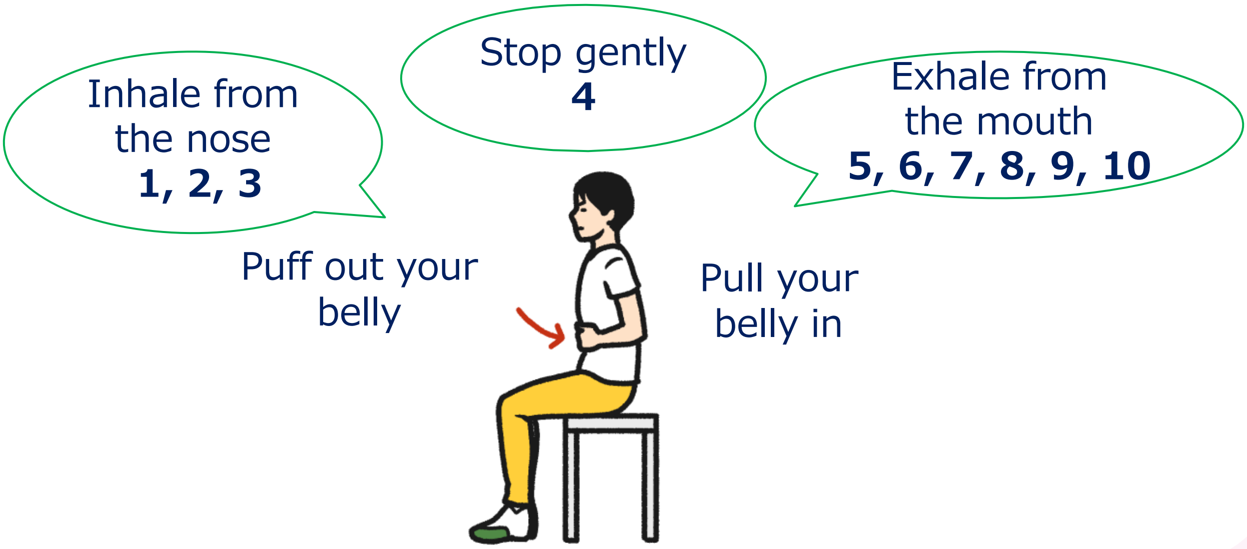

Progressive muscle relaxation

Relaxation can be achieved by putting pressure on different parts of the body and then loosening them. It is recommended before going to bed or on waking up at night.

Hands

Squeeze tightly (5 s) ⇒ Spread slowly (10 s)

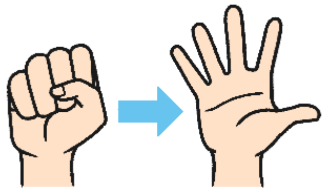

Arms

Arms should be bent as if making a bump, then they should be squeezed tightly at the sides (5 s)
⇒ Relax (10 s)

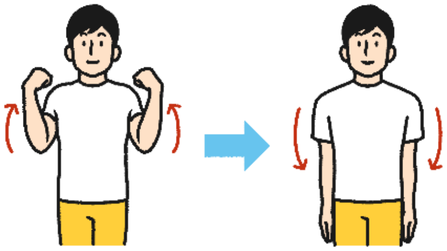

Shoulders

Both shoulders should be raised firmly and tensed close to the ears (5 s)
⇒ Should be pulled out (10 s)

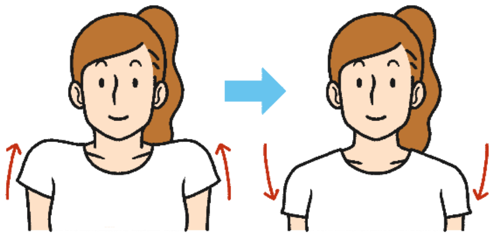

Face

The eyes and mouth should be closed tightly, with biting on the back teeth (5 s) ⇒ The mouth should be opened with a pout (10 s)

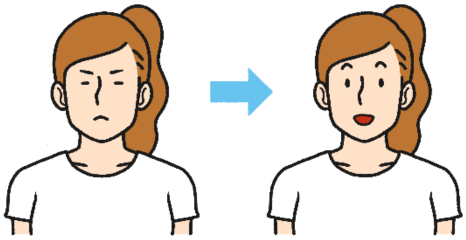

Progressive muscle relaxation

succession

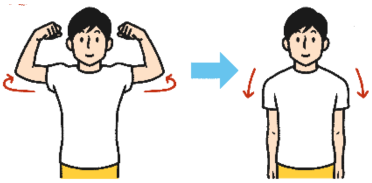

Back

The arms should be spread outwards and the shoulder blades should be pulled together (5 s)⇒ Pull out (10 s)

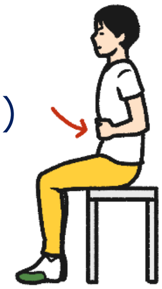

Abdomen

The abdomen should be squeezed (5 s)
⇒ Pull it out (10 s)

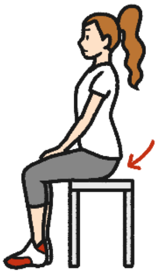

Buttocks

The buttocks should be squeezed as if tightening them (5 s)
⇒ Pull it out (10 s)

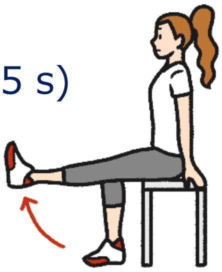

Legs

The complete leg should be gently tensed (5 s)
⇒ Pull it out (10 s)

More information about muscle relaxation techniques can be found on the Ministry of Education, Culture, Sports, Science and Technology website.

https://www.mext.go.jp/a_menu/shotou/clarinet/002/003/010/004.htm

Visualisation

Visualisation is a method for improving your mood by imagining the situations in which you feel most relaxed. For example, you could imagine that you are relaxing on a porch, looking at the view and enjoying the smell of tea, or lying in the spring grass in the soft rays of the sun.

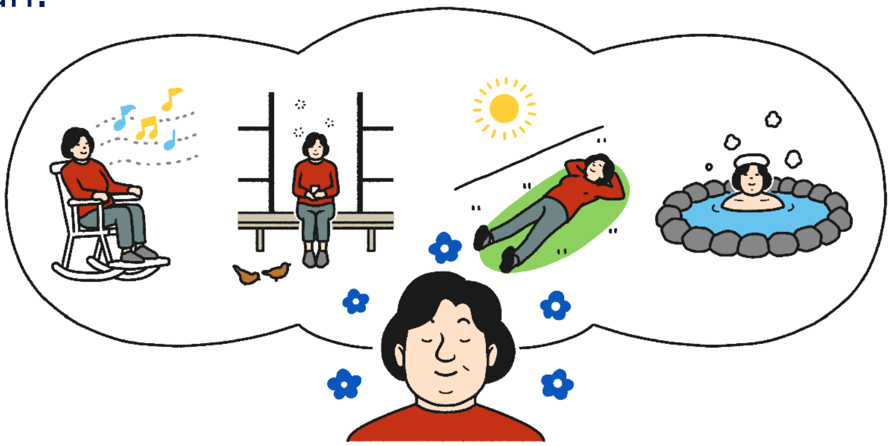

It may be easier to do this after performing other relaxation activities. Additionally, it may be better to do this with a therapist at first, because it can be easier to visualise with your eyes closed while someone is talking to you.

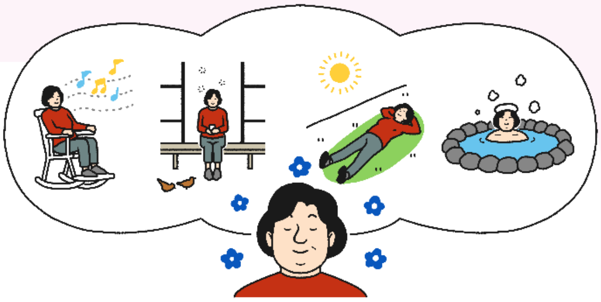

Visualisation

Talking to yourself is another way to relax. You can try doing this during breaks or before going to bed. The key point is to always perform an elimination movement when you finish performing the activity. It is recommended that this is also performed with a therapist at first.

1. Get into a comfortable position in a calm place where you can relax easily.

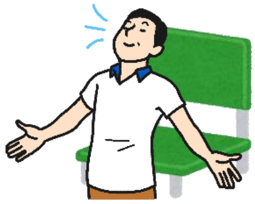

2. Close your eyes and take a deep breath.
3. Say the following words in your mind, in order: "I feel calm...", "My arm feels heavy...", "My arm feels warm...".
4. Think of a place that is warm and calming, such as a hot spring, or a hot sun bed.
5. When the sensations of heaviness and warmth become clear, gradually extend your attention to the whole body, from the right arm to both arms to both feet, and feel the sensations.
6. Finish with an elimination movement: (1) Grab your hands and release 2-3 times; (2) Bend and stretch both elbows slowly; and (3) Stretch up tall and stretch.

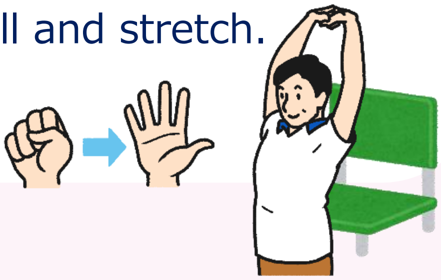

3. Stress coping

Daily stress coping can help prevent anxiety and tension, and can also help you recover when you feel anxious. Many people use the following activities to help them cope with stress. It can be helpful to build up a repertoire of healing activities that work for you.

【Activities】

- Eating out at a restaurant
- Drinking juice
- Buying a cake to take home
- Buying clothes
- Buying manga
- Renting DVDs
- Taking a different route than usual
- Going for a walk
- Doing some light exercise
- Going to a batting practice cage
- Wearing brightly coloured clothes
- Eating meals with your favourite dish wear/cutlery
- Watching TV or YouTube
- Watching sports
- Speaking with others
- Saying “no” from time to time
- Listening carefully to others
- Distancing yourself from people you don’t like spending time with
- Complaining to a plush toy
- Saying "thank you" a lot
- Replying to emails you haven’t responded to
- Calling or texting friends you don’t see regularly
- Writing about stress in a diary
- Tearing up old papers or magazines
- Organising and lightening your bag
- Organising your desk
- Polishing your shoes
- Drawing a picture
- Humming a song
- Buying a houseplant
- Arranging flowers

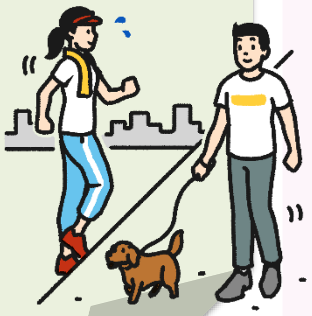

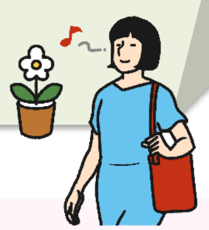

【Rest】

- Take a leisurely bath (with bath salts)
- Aromatherapy or burning incense
- Changing your mood by listening to your favourite music
- Taking a deep breath
- Lying down in the garden or on the grass
- Going to the rooftop after lunch and looking at the sky
- Taking a nap
- Taking a break from time to time
- Taking a few days off work

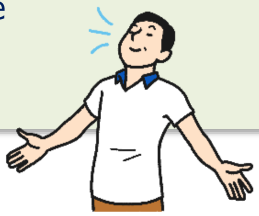

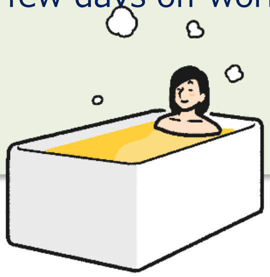

【Five senses】

- Getting a foot massage
- Doing yoga or stretching
- Jogging
- Swimming, walking in the water
- Playing with pets
- Playing games
- Browsing the internet
- Having your favourite food and drink
- Visiting museums or gardens
- Stargazing
- Looking at old photos
- Reading
- Travelling
- Cooking
- Fishing
- Making ceramics
- Playing go or shogi (Japanese chess)
- Playing musical instruments
- Watching live music
- Doing handicrafts or knitting
- Going skiing or snowboarding
- Doing outdoor activities
- Gardening
- Driving
- Shopping
- Visiting a zoo or aquarium
- Using a new perfume
- Visiting a planetarium
- Doing karaoke
- Watching a film

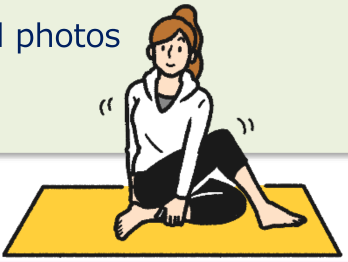

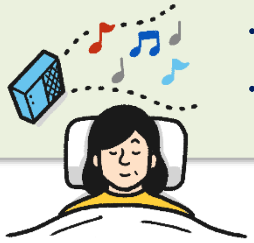

Appendix 4

Frequently asked questions and answers about anxiolytics

● Will I not be able to stop anxiolytics?

You will not be able to stop within a short period of time after you start using the drug. If you are currently using the drug and your anxiety is improving, you can gradually reduce the dosage to decrease or stop the drug (see page 13). If you stop at your own discretion, it may worsen. Please consult your doctor before reducing the dosage.

● Isn't the effect of the medication getting weaker and the dosage increasing gradually?

Depending on the type, there are some medications that tend to weaken the effect and others that do not. When the effect becomes weaker, change the medicine type or consider other treatments. If you feel that the effect has weakened, kindly inform.

● I am worried because I have heard that some people have died from taking too much.

The common anxiolytics that are currently prescribed, as described on page *, are highly safe. However, if taken in large amounts at once or with alcohol, they can cause serious side effects such as respiratory depression. Thus, always use them as per the doctor's direction.

● I am worried about taking other medication together.

Anxiolytics are known to interact with antidepressants and some drugs for lifestyle-related diseases. If you start taking other medications, kindly inform us. When you go to the hospital, carry your medication notebook and show it to your doctor.

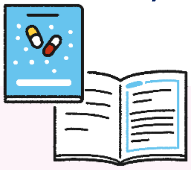

● I have heard that it can cause dementia.

There are some reports that long-term use increases the risk of developing dementia, while others say it does not, and no conclusion has been reached. Long-term use is not recommended for people who have already been diagnosed with dementia or who are at high risk of dementia.

● Is there any effect on the foetus?

We will consider the effects of the medication and the timing of the pregnancy to determine whether to continue or stop. The effects of anxiolytics on the foetus are still being studied. Once you find out that you are pregnant, we will get the latest information and discuss it with you.

Comments

(A note regarding the questions for the doctor should be prepared)

-
-
-
-

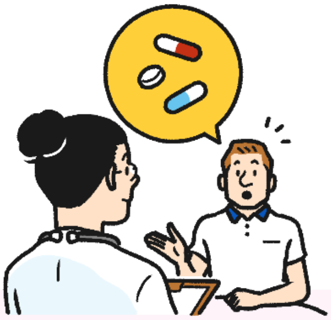

Conclusion

●To determine the right coping and treatment methods that are best for you

Each treatment option has its pros and cons. This decision aid is designed to help you understand these options well, discuss them with your healthcare provider while determining what is important to you, and make the choices that are right for you.

● Development of the decision aid

This decision aid was developed based on the responses and opinions of people who had been taking medication treatment for insomnia. The decision aid has also been checked by psychiatric specialists. In addition, this aid was developed by research grants from the Ministry of Health, Labor and Welfare of Japan (19GC1012).

● Updating the decision aid

This decision aid will be reviewed and updated as necessary.

The information provided here is intended to guide you in identifying the right coping or treatment method for you while you are consulting with a healthcare professional; however, this decision aid is not intended to replace the advice provided by the healthcare professional.

文献

- Takeshima M, Otsubo T, Funada D, et al.: Does cognitive behavioral therapy for anxiety disorders assist the discontinuation of benzodiazepines among patients with anxiety disorders? A systematic review and meta-analysis. Psychiatry Clin Neurosci. 2021, 75(4):119-127. doi: 10.1111/pcn.13195. (16ﾍﾟｰｼﾞ)

Develop by Anxiolytics Group in Psychotropic Drugs Exit Strategy Manual Research Group

Created: July 2022
Scheduled to be updated: July 2024

This decision aid is based on the Guidelines for the Appropriate Use and Withdrawal of Sleep Medication and the Guidelines for Psychotropic Drug Discontinuation Strategies

©2022, Psychotropic Drugs Exit Strategy Manual Research Group

継続　減薬　中止

今後の治療法を一緒に決めるための手引き

抗不安薬

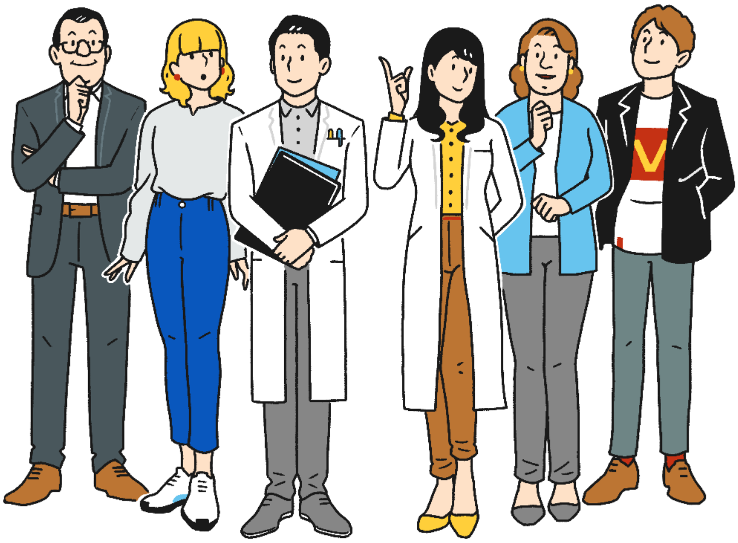

この手引きは**「向精神薬の出口戦略ガイドライン」**にもとづいて作成されています

もくじ

この手引きについて／手引きの使い方 1

不安症とは 4

この先の治療の選択肢 6

選択肢1『抗不安薬の使用を継続する』 7

選択肢2『抗不安薬を減量・中止する』 8

抗不安薬を減量・中止する場合の この先の治療の選択肢 12

選択肢1『漸減法（ぜんげんほう）を用いて抗不安薬を減量・中止する』 13

選択肢2『漸減法と認知行動療法（にんちこうどうりょうほう）を併用して抗不安薬を減量・中止する』 14

付録1 抗不安薬一覧 19

付録2 それぞれの不安症について 20

付録3 不安の軽減のためにできること 23

付録4 抗不安薬に関するよくある質問と回答 31

この手引きについて

　この手引きは、不安症（パニック症／広場恐怖、社交不安症、全般不安症）でベンゾジアゼピン系抗不安薬（以下、抗不安薬）を使用中の方が、この先の治療について医療者と一緒に決めるためのものです。抗不安薬を長期間漫然と使用することは、副作用や依存の問題から望ましくありません。

　不安症に対しては、主に選択的セロトニン再取り込み阻害薬（SSRI）とセロトニン・ノルアドレナリン再取り込み阻害薬（SNRI）が使われ、同時に、認知行動療法も少しずつ取り入れられるようになっています。中には、SSRIやSNRIに加えて、抗不安薬を併用している、あるいは、抗不安薬のみで経過をみている方がいらっしゃると思います。

　　そこで、**抗不安薬を継続するか、減量・中止するか、**減量・中止する場合は、**認知行動療法を併用するかどうか**、それぞれの長所・短所を比較しながら、自分に合った方法を医療者と一緒に選ぶために、この手引きを作成しました。

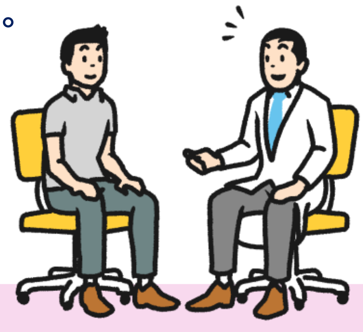

この手引きについて つづき

【この手引きの対象になる方】

・抗不安薬※ を使用中で、不安症の症状が概ね改善していて、日中の心身の状態もよい方

おもに抗不安薬のみを使用されている方が対象となりますが、抗不安薬以外の向精神薬を併用されている方も対象となりえます。

【この手引きの対象とならない方】

・不安症の症状が十分に改善していない方

・日中の心身の状態がよくない方

※ ここで取り上げている抗不安薬の一覧を 巻末の **付録1**（19ﾍﾟｰｼﾞ） に示しました。ご自分が使用している薬があるか確認ください。

ご自身がこの手引きの対象となるかわからない方は
医師に相談しましょう

この手引きの使い方

　この手引きは、医療者と話し合いながら、この先の治療法を選ぶためのものです。この手引きを自宅に持ち帰ってよく読み、ご家族などに相談するなどし、十分に検討しながら、今後の治療の方針を一緒に考えていきます。

手引きをよく読みます
○をつけたりメモ欄に
記入したりします

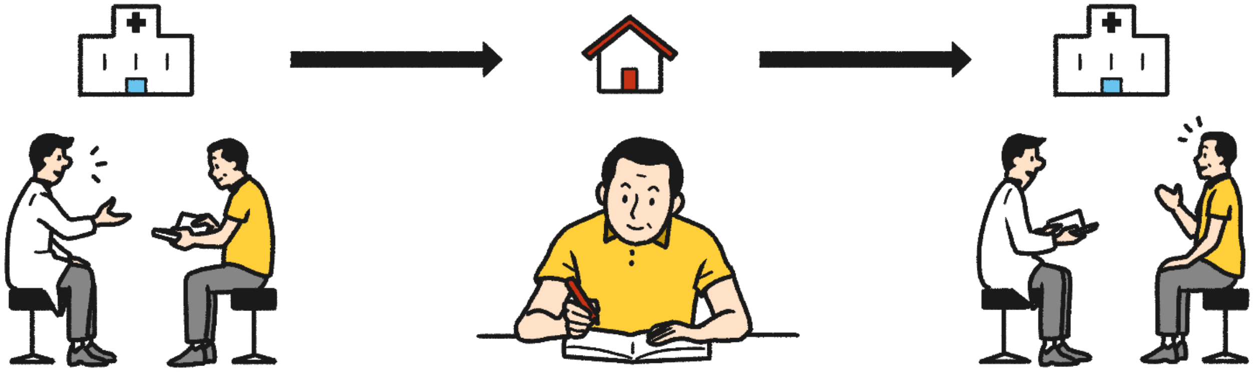

診察で

・現在の状態
・治療の選択肢
　について確認します

診察で

・○ をつけた項目や
　メモした内容について
　話し合います
・今後の方針を決めます

※一度で決まらない場合は
また持ち帰って検討します

不安症とは

　代表的な不安症には、**パニック症/広場恐怖**、**社交不安症**、**全般不安症** があります。それぞれの不安症で、症状の種類や起こり方が異なります。

　パニック症 は、突然のパニック発作や『また不安が起こるのではないか』という予期不安と特定の場所や状況を避けるようになる広場恐怖を特徴とする不安症です。

　社交不安症 は、他人から注目を浴びる可能性のある社会的状況に対して、強い不安や恐怖が生じる不安症です。

　全般不安症 は、慢性的にコントロール出来ない心配のせいで日常生活がままならなくなっている不安症のことをいいます。

　ご自身がどの不安症に当たるのかは、医師によく聞いてみましょう。それぞれの不安症に関しては、巻末の **付録2** 20ページ に示してあります。参考にしてください。

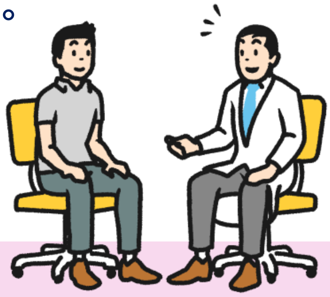

不安症とは つづき

　日常生活の工夫だけで不安症が改善しない場合、SSRI か SNRI の単剤治療が勧められています。中には、それに加えて抗不安薬（ベンゾジアゼピン系）が使用される場合があります。抗不安薬は副作用などの問題から、長期間漫然と使用することは望ましくありません。また、抗不安薬による依存や、中止時の離脱症状（りだつしょうじょう）の問題もあります。とくに、SSRI や SNRI は使用されず、抗不安薬のみが使用されている場合には注意が必要です。

　不安症状が改善し、日中の心身の状態も安定するようになったら、抗不安薬の使用を継続するか、中止に向けた減量をするか検討していきます。

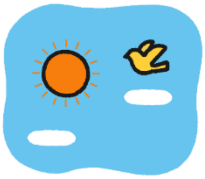

　つぎは、不安症状が改善し、日中の心身の状態も安定している場合の、この先の治療の選択肢をみていきます ☞

この先の治療の選択肢

ステップ 1 各選択肢の内容を理解しましょう

選択肢1 『抗不安薬の使用を継続する』

この選択肢を選んだ場合に行うこと

・抗不安薬の使用を継続する ➡ **7**ページ

・日常生活で不安の軽減のためにできることに取り組む
➡ 巻末 **付録3** **23**ページ

・SSRI や SNRI を併用している場合は、それを継続する

選択肢2 『抗不安薬を減量・中止する』

この選択肢を選んだ場合に行うこと

・抗不安薬を減量・中止する ➡ **8**ページ

・日常生活で不安の軽減のためにできることに取り組む
➡ 巻末 **付録3** **23**ページ

・SSRI や SNRI を併用している場合は、それを継続する

選択肢1 『抗不安薬の使用を継続する』

抗不安薬を継続する長所・短所を示しておきます。

【長所】

抗不安薬を継続することによって

・不安症状が改善した状態をこのまま維持できる

・いまの生活や心身の安定した状態をこのまま維持できる

【短所】

抗不安薬を継続することによって

・抗不安薬による副作用（転倒、眠気、認知機能の低下、健忘など）や依存が起こる可能性がある

・他の薬との飲み合わせに注意する必要がある

・妊娠、授乳に影響する場合がある

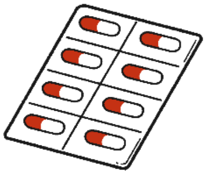

選択肢2 『抗不安薬を減量・中止する』

抗不安薬を、長期間漫然と使用することは望ましくありません。不安症状が改善し通常の生活が可能で、日中の心身の状態が安定していれば、減量・中止を検討できるタイミングといえます。

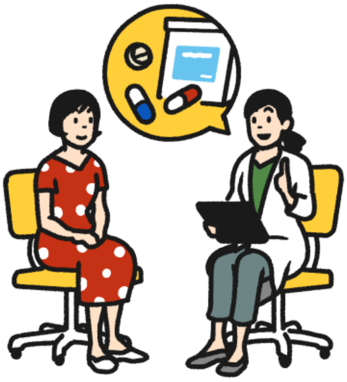

つぎは、『抗不安薬を継続する』『抗不安薬を減量・中止する』それぞれの選択肢の長所・短所を比較してみましょう ☞

各選択肢の長所・短所

ステップ 2 各選択肢の長所・短所を比較してみます

	選択肢1<br>**『抗不安薬を継続する』**<br>・抗不安薬の使用を継続する<br>・日常生活で不安の改善のためにできることに取り組む⇒付録3,23ﾍﾟｰｼﾞ	選択肢2<br>**『抗不安薬を減量・中止する』**<br>・抗不安薬を減量・中止する<br>・日常生活で不安の改善のためにできることに取り組む⇒付録3,23ﾍﾟｰｼﾞ
**長所** ☺	・現在の安定した状態を維持できる<br>・離脱症状※ に悩まされない	・やめられなくなる不安がなくなる<br>・副作用※※ や依存が生じる可能性に悩まされなくなる
**短所** ☹	・やめられなくなる不安が続く<br>・副作用※※ や依存が生じる可能性に悩まされることがある	・不安症状や日中の心身の状態が悪化する可能性がある<br>・やめたときに離脱症状※ が出ることがある

※離脱症状：嘔気、発汗、めまい、不安、消化器症状 など

※※副作用：転倒リスク、眠気、認知機能の低下、健忘 など

自分にとって重要なこと

ステップ 3 あなたにとって重要なことを整理します

以下に、各選択肢を選ぶおもな理由をあげました。それぞれあなたにとってどのくらい重要ですか？あてはまる数字に○をつけ、重みづけをしてみましょう。

選択肢1『抗不安薬の使用を 継続する』理由

	重要でない					重要である
不安症状の改善した状態がこのまま続く	0	1	2	3	4	5
安定した生活や心身の状態がこのまま続く	0	1	2	3	4	5
その他（自由に記載）						
・	0	1	2	3	4	5
・	0	1	2	3	4	5

選択肢2『抗不安薬を 減量・中止する』理由

	重要でない					重要である
やめられなくなる心配がない	0	1	2	3	4	5
副作用や依存に悩まされない	0	1	2	3	4	5
その他（自由に記載）						
・	0	1	2	3	4	5
・	0	1	2	3	4	5

診察で話し合うための準備

ステップ 4 医師との話し合いにむけた準備をします

あなたの考えや重みづけをもとに、**選択肢1『抗不安薬を継続する』** **選択肢2『抗不安薬を減量・中止する』**について診察で話し合います。

疑問や気になったことなど、今の気持ちや考えを書いておきましょう

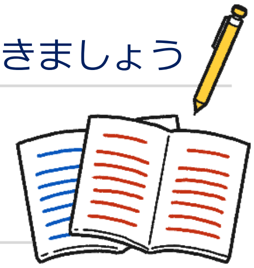

『抗不安薬を減量・中止する』ことを希望する場合は、つぎに進みます☞

『抗不安薬を減量・中止する』場合の

この先の治療の選択肢

ステップ 1 各選択肢の内容を理解しましょう

選択肢1 『漸減法(ぜんげんほう)を用いて減量・中止する』

この選択肢を選んだ場合に行うこと

・日常生活で不安の軽減のためにできることに取り組む

➡ 巻末 付録3 23ページ

・漸減法を用いて抗不安薬を減量・中止する ➡ 13ページ

選択肢2 『漸減法と認知行動療法を併用して減量・中止する』

この選択肢を選んだ場合に行うこと

・日常生活で不安の軽減のためにできることに取り組む

➡ 巻末 付録3 23ページ

・漸減法を用いて抗不安薬を減量・中止する ➡ 13ページ

・漸減法と認知行動療法を併用して減量・中止する➡ 14ページ

選択肢1 『漸減法（ぜんげんほう）を用いて減量・中止する』

抗不安薬は、突然中止すると、薬を使い始める前よりも強い不安が現れることがあります。これを反跳性不安（はんちょうせいふあん）と言います。必ず医師と相談しながら少しずつ減らし、最終的に薬を使用しなくても生活できるようになるのを目指します。

【薬の減らし方・漸減法】

- 2〜4週間かけて1/2錠以下ずつゆっくり減らします
- はじめの数日は反跳性不安を自覚することもありますが、徐々に改善します
- 不安症状が悪化したら1段階戻ります

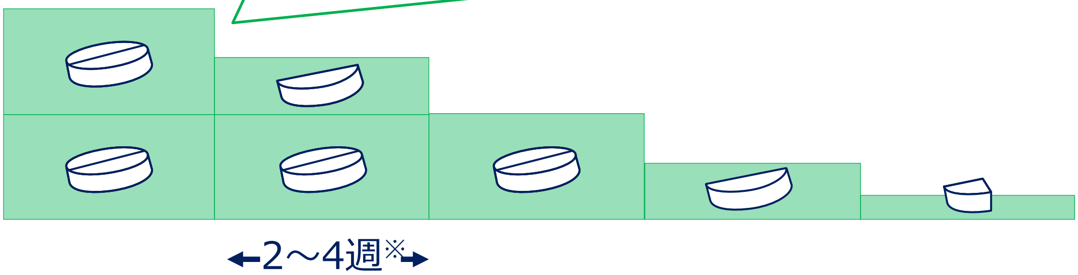

※期間は目安です。医師と相談しながらゆっくりと減らしていきます

中止後、使用していた時と比べ、違和感があることがありますが、生活に支障がなく、思うように行動ができていれば問題ありません。

選択肢2 『漸減法と認知行動療法を併用して減量・中止する』

不安症に対する認知行動療法とは、不安を感じやすい認知（考え）や生活習慣の改善をはかり、リラクセーションの方法を身につけながら、不安の改善をめざす治療法です。

訓練を受けた専門家（医師や看護師、心理士など）のもと、週1回50~90分、合計12~16回を目安に行います。
各不安症の認知行動療法の詳しい解説は、日本不安症学会のホームページを参照ください。

➡ 日本不安症学会認知行動療法マニュアル jpsad.jp/manual.php

認知行動療法は抗不安薬のような即効性はありませんが、抗不安薬と同等に不安に効果があるとされています。
また、減量・中止する際に実施すると、中止しやすくなるとされています。一方で、現在のところ、実施施設がまだ限られていますが、パニック症と社交不安症は医療保険の適用となる場合もあります。

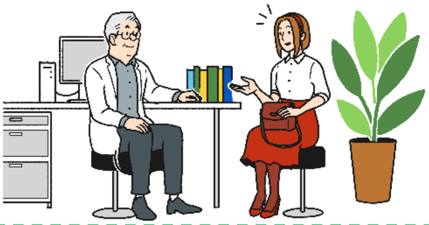

つぎは、『漸減法を用いて減量・中止する』『認知行動療法を併用して減量・中止する』それぞれの選択肢の長所・短所を比較してみましょう ☞

各選択肢の長所・短所

ステップ 2 各選択肢の長所・短所を比較してみます

	**選択肢1**<br>**『漸減法を用いて減量・中止する』**<br>・漸減法を使って抗不安薬を減量・中止する<br>・日常生活で不安の軽減のためにできることに取り組む⇒付録3,23ﾍﾟｰｼﾞ	**選択肢2**<br>**『漸減法と認知行動療法を併用して減量・中止する』**<br>・漸減法を使って抗不安薬を減量・中止する<br>・認知行動療法を併用する<br>・日常生活で不安の軽減のためにできることに取り組む⇒付録3,23ﾍﾟｰｼﾞ
**長所** ☺	・現在の担当医のもとでできる<br>・新たな費用はかからず、保険診療の範囲内でできる<br>・診療以外の時間や労力を必要としない	漸減法のみと比べ、<br>・抗不安薬をより減量・中止しやすくなる<br>・抗不安薬を減量・中止後の不安症状の悪化を認めにくい可能性がある
**短所** ☹	認知行動療法を併用する場合と比べ、<br>・抗不安薬を減量・中止しにくいことがある<br>・不安症状が悪化する場合がある	認知行動療法は<br>・他の医療機関への通院が必要になる可能性がある<br>・新たに費用がかかり、保険診療の範囲内でできない可能性がある<br>・効果を実感するのに時間や労力を必要とする場合がある

各選択肢を選んだ結果

ステップ 3 各選択肢を選んだ結果を比較してみます

抗不安薬を使用中の人が各選択肢を選んだ場合、どの位の割合で抗不安薬を中止できるか、我々の推定データを示しました。
顔1つが1人を表し、100人中何人が抗不安薬を中止できるか示しています。

	**選択肢1**<br>**『漸減法を用いて減量・中止する』**	**選択肢2**<br>**『漸減法と認知行動療法を併用して減量・中止する』**
3ヶ月後	漸減法を用いて減量・中止すると、3ヶ月後 100人中33人が抗不安薬を減量・中止できる<br>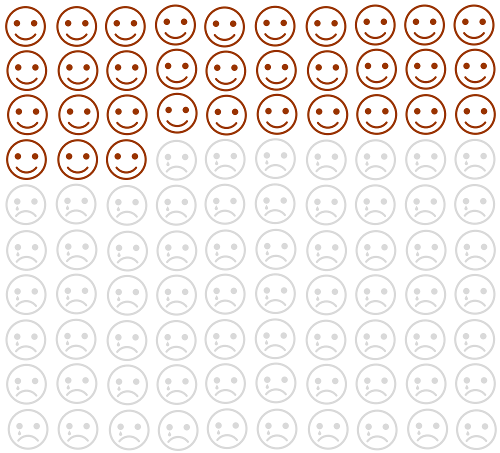	漸減法を用いて減量・中止し、さらに認知行動療法を併用すると、3ヶ月後 100人中64人が抗不安薬を減量・中止できる<br>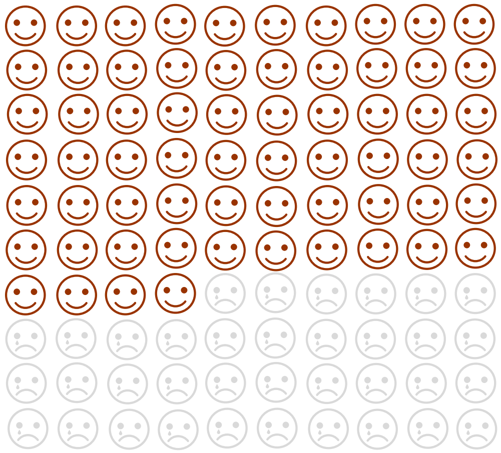
6〜12ヶ月後	漸減法を用いて減量・中止すると、6〜12ヶ月後 100人中31人が抗不安薬を減量・中止できる<br>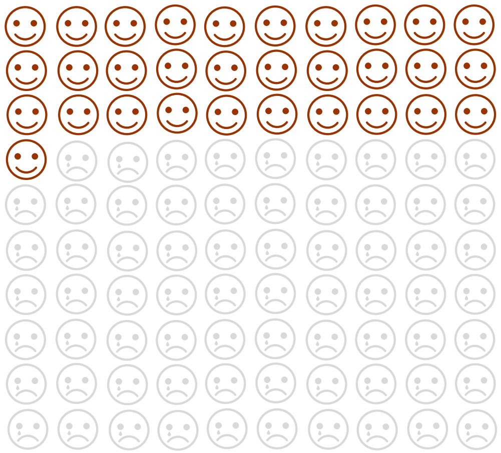	漸減法を用いて減量・中止し、さらに認知行動療法を併用すると、6〜12ヶ月後 100人中67人 が抗不安薬を減量・中止できる<br>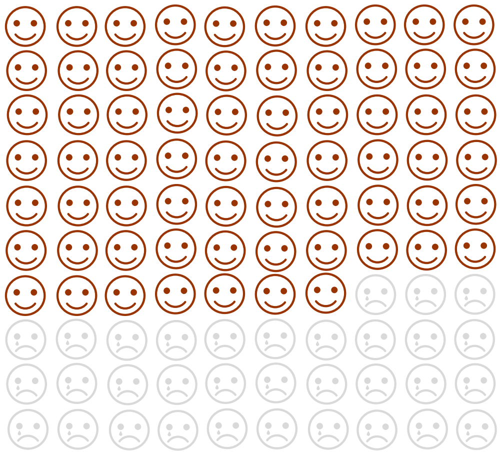

自分にとって重要なこと

ステップ 4 あなたにとって重要なことを整理します

以下に、各選択肢を選ぶおもな理由をあげました。それぞれあなたにとってどのくらい重要ですか？あてはまる数字に〇をつけ、重みづけをしてみましょう。

選択肢1『漸減法を用いて 減量・中止する』理由

	重要でない					重要である
現在の担当医のもとでできる	0	1	2	3	4	5
保険診療の範囲内でできる	0	1	2	3	4	5
その他（自由に記載）						
・	0	1	2	3	4	5
・	0	1	2	3	4	5

選択肢2『漸減法と認知行動療法を併用して減量・中止する』理由

	重要でない					重要である
薬が減らしやすくなる	0	1	2	3	4	5
不安が生じにくい	0	1	2	3	4	5
その他（自由に記載）						
・	0	1	2	3	4	5
・	0	1	2	3	4	5

診察で話し合うための準備

ステップ 5 医師との話し合いにむけた準備をします

　あなたの考えや重みづけをもとに、**選択肢1『漸減法を用いて減量・中止する』選択肢2『認知行動療法を併用して減量・中止する』**について、つぎの診察で話し合います。

疑問や気になったことなど、今の気持ちや考えを書いておきましょう

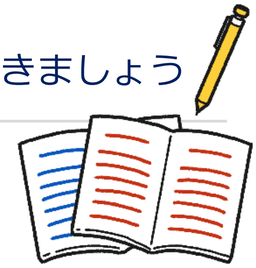

付録1　抗不安薬一覧

付録2　それぞれの不安症について

付録3　不安の軽減のためにできること

付録4　抗不安薬に関するよくある質問と回答

付録1　抗不安薬一覧

おもな抗不安薬を表にしました。表を参考に、使用中の薬の特徴について、医師と一緒に確認しましょう。

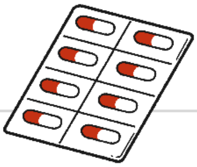

短い ↑　効く時間　↓ 長い

	一般名【代表的な商品名】
短時間作用型	エチゾラム【デパス®】<br>クロチアゼパム【リーゼ®】<br>フルタゾラム【コレミナール®】
中間作用型	ロラゼパム【ワイパックス®】<br>アルプラゾラム【コンスタン®/ソラナックス®】<br>フルジアゼパム【エリスパン®】<br>ブロマゼパム【レキソタン®/セラニン®】<br>ジアゼパム【セルシン®/ホリゾン®】
長時間作用型	クロキサゾラム【セパゾン®】<br>クロルジアゼポキシド【コントール®】<br>メダゼパム【レスミット®】<br>フルトプラゼパム【レスタス®】
超長時間作用型	ロフラゼプ酸エチル【メイラックス®】<br>メキサゾラム【メレックス®】<br>オキサゾラム【セレナール®】

利点 ☺ 不安が軽減する、値段が比較的安い、
[作用時間の短いものは] 蓄積を生じにくい
[作用時間の長いものは] 使用回数を削減できる

欠点 ☹ 徐々に効き目が薄れる、副作用（転倒、眠気、認知機能の低下、健忘等）がでる可能性がある、やめたときの離脱症状（吐き気・嘔吐・発汗・めまい・不安等）、依存の問題がある
[作用時間の短いものは] 連用後に中断すると離脱症状を自覚しやすい
[作用時間の長いものは] 連用によって体内蓄積を起こす恐れがある、離脱症状の自覚は少ない

付録2 それぞれの不安症について

パニック症／広場恐怖

パニック症／広場恐怖とは

パニック症とは、不安の出現が特徴的な精神疾患である不安症の一つです。ある限定した時間内に死の恐怖や、発狂してしまうのではないかという恐怖とともに、動機、呼吸困難、発汗、めまい、震え、窒息感といった体の症状が出現し、これをパニック発作と呼んでいます。パニック症は、このパニック発作が突発的かつ反復性に出現することが特徴です。パニック発作が再び出現するのを恐れ、不安にかられる『予期不安』や、パニック発作が起きたときに助けを求められない、または逃げ出すことができない状況や場所を恐怖し、回避する状態である『広場恐怖』を合併します。わが国での有病率は約1.7～3.3%で、女性の方が約2~3倍多いと考えられています。女性の場合は20～30代、50~60代が多く、男性は30代以降では減少するといわれています。

パニック症／広場恐怖の治療

パニック症の治療法には、薬物療法と認知行動療法を含む精神療法があります。薬物療法については、現在は第一選択薬として抗うつ薬のSSRIが推奨されていますが、効果発現に2～6週と時間がかかるため、短期的には即効性のあるベンゾジアゼピン系抗不安薬が用いられることがあります。ただし、ベンゾジアゼピン系抗不安薬は依存や乱用の問題があるため、注意して用いる必要があり、長期使用はすすめられていません。

非薬物療法については、疾患について正しく理解するための心理教育や認知行動療法があります。認知行動療法は薬物に効果がない方にも有効なことがあります。他に、パニック発作を起こしやすくするストレスや嗜好品（刺激物）を避けるなど、生活上の工夫も大切です。

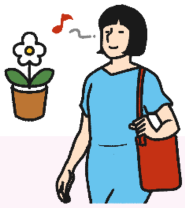

付録2 それぞれの不安症について

社交不安症

社交不安症とは

社交不安症とは、他人から注目を浴びる可能性のある社会的状況に対して、強い不安や恐怖が生じる状態です。『自分のふるまいや自分が不安がっていることにより、恥をかいてしまうのではないか、他の人に拒絶されるのではないか』など否定的な評価をされることを恐れるあまり、社交的な集まりや発表などの社会的状況を避けるようになり、日常生活や社会生活に支障をきたします。社交不安症と診断するには、これらの症状が半年以上続いている必要があります。

発症年齢は早く、10歳代がピークといわれています。症状は長く続くことが一般的です。

社交不安症の治療

治療法は大きく分けて薬物療法と認知行動療法を含む精神療法があり、短期的な効果は同程度といわれています。日本では薬物療法として抗うつ薬や抗不安薬がよく用いられていますが、抗うつ薬が第一選択薬です。抗不安薬は依存などの問題もあるため、治療を始めたばかりの症状の重い方や他の治療で効果が得られなかった方で、症状が重く、迅速な症状の緩和が必要な方に対して、短期的な使用が好ましいといわれています。

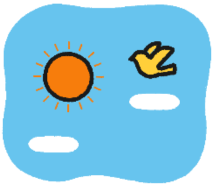

付録2 それぞれの不安症について

全般不安症

全般不安症とは

　全般不安症は、慢性的にコントロール出来ない『心配』（あることないことを想像して強い危機感を持って心配する）を中心症状とする病気です。心配によって、十分眠れなくなったり、筋肉が緊張して凝ったりなどの身体の症状や、集中できなくなることで、深刻な社会的・職業的機能上の問題を起こしたり、他の精神疾患に発展したり、自殺の危険性が上がったりすると考えられています。

　しかし、『心配』という症状は、うつ病や他の不安症、病気不安症（心気症）などでもみられるので、どうしても全般不安症の診断が後回しになる傾向があります。

全般不安症の治療

　全般不安症の治療のおもなものは、薬物療法と認知行動療法を含む精神療法です。

　全般不安症に対する第一選択薬は、SSRIかSNRIを中心とした抗うつ薬です。他に、（ベンゾジアゼピン系）抗不安薬なども使われますが、抗不安薬は副作用や依存の問題から、抗うつ薬の効果発現までの治療初期（1ヵ月以内）に限定した使用が勧められています。

　薬物療法と同等に重要な治療として精神療法があります。基本は受容と共感といういわゆる小精神療法的アプローチが重要で、それを踏まえた上で、必要な方には認知行動療法が勧められます。

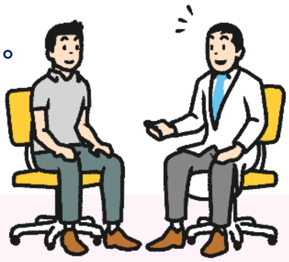

付録3　不安の軽減のためにできること

　不安や緊張は、日常生活の工夫においても和らげる方法があります。

1. 基本事項

1. 規則正しい生活リズムを心がけ、寝る時刻と起きる時刻を一定にし、食事も三食きちんととります

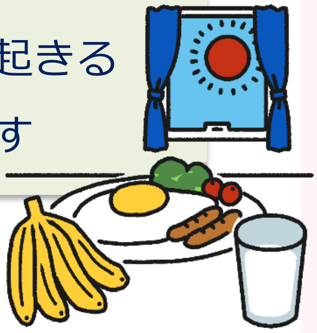

2. お酒の飲みすぎ、タバコの吸いすぎ、カフェインなどの刺激物のとりすぎに注意します

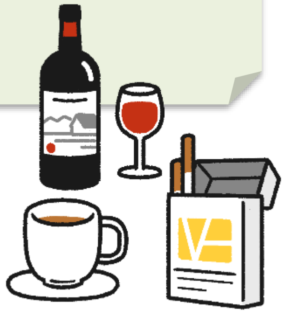

3. 過労に注意し、適度な運動を心がけます

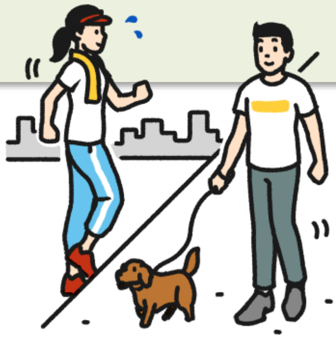

2. リラクセーション法

　不安が強い状態というのは、普段から呼吸が浅くなったり、筋肉の緊張が高かったりするものです。日頃からこころや体をリラックスさせておくのも不安を和らげることにつながります。代表的なものをご紹介します。

呼吸法

　鼻から吸って、止めて、口から吐く、という呼吸です。秒数はご自分で調節して構いません。ポイントは 、
①吐くほうを長くゆっくり ②腹式呼吸 ③息を吐く時には不安や緊張、ストレスなどを体から吐き出すイメージを持つ、の３つです。1日に２～３回程度の日課にすると良いでしょう。

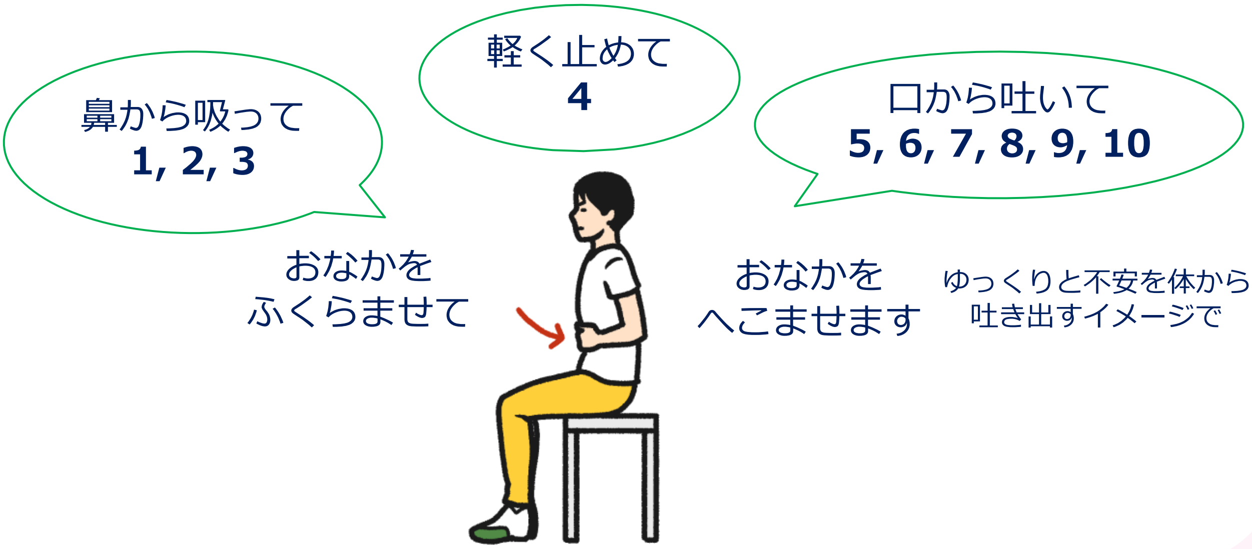

きんしかんほう
筋弛緩法

体のいろいろな場所に力を入れて、それを緩めることでリラックスしていきます。初めはなかなか力をスッと抜くのが難しいものですが、徐々に慣れてきます。

両手

ギューっと握って（5秒）⇒ ゆっくり広げます（10秒）

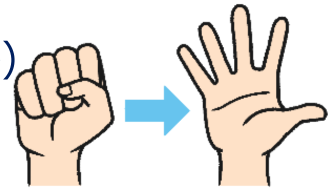

両腕

力こぶを作るように腕を曲げ、脇を締めてギューッと力を入れ（5秒）
⇒ ストンと抜きます（10秒）

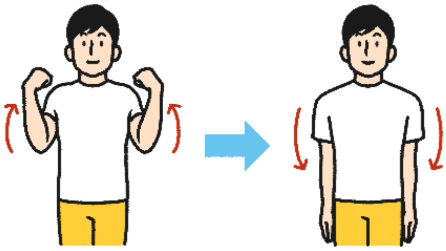

両肩

両肩をグッと上げ耳まで近づけて緊張させて（5秒）
⇒ ストンと抜きます（10秒）

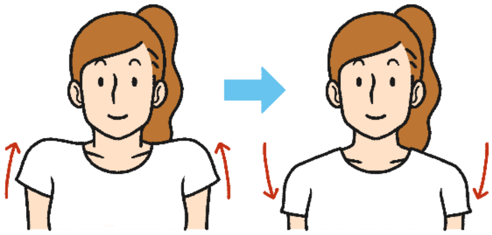

顔

目と口をギューっとつぶって、奥歯を噛みしめて（5秒）
⇒ ポカンと口をあけます（10秒）

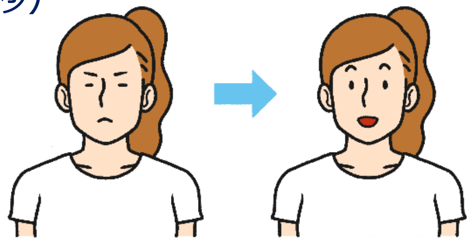

筋弛緩法 つづき

背中
腕をグーッと外に広げて肩甲骨を引き付けて（5秒）
⇒ ストンと抜きます（10秒）

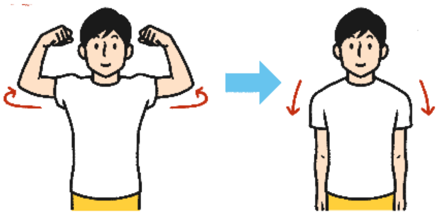

おなか
おなかをへこませて、おなかに力を入れて（5秒）
⇒ ストンと抜きます（10秒）

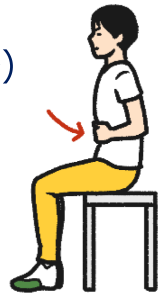

おしり
おしりの穴を引き締めるようにギューッと力を入れて（5秒）
⇒ ストンと抜きます（10秒）

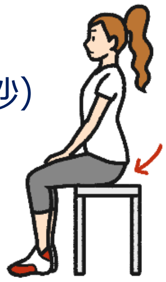

脚
足全体にグーっと力を入れて緊張させて（5秒）
⇒ ストンと抜きます（10秒）

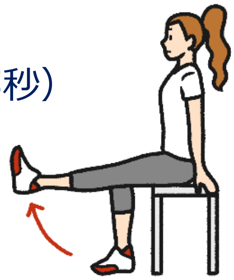

筋弛緩法についてさらに詳しく・・・
文部科学省ホームページ
https://www.mext.go.jp/a_menu/shotou/clarinet/002/003/010/004.htm

イメージ法

　自分が最もリラックスできる場面を想像して気分を軽くする方法です。例えば、ポカポカした縁側でのんびりと外を眺めてお茶の香りを楽しんでいるとか、春の草むらで太陽の柔らかい日差しを浴びながら寝転がっている などです。

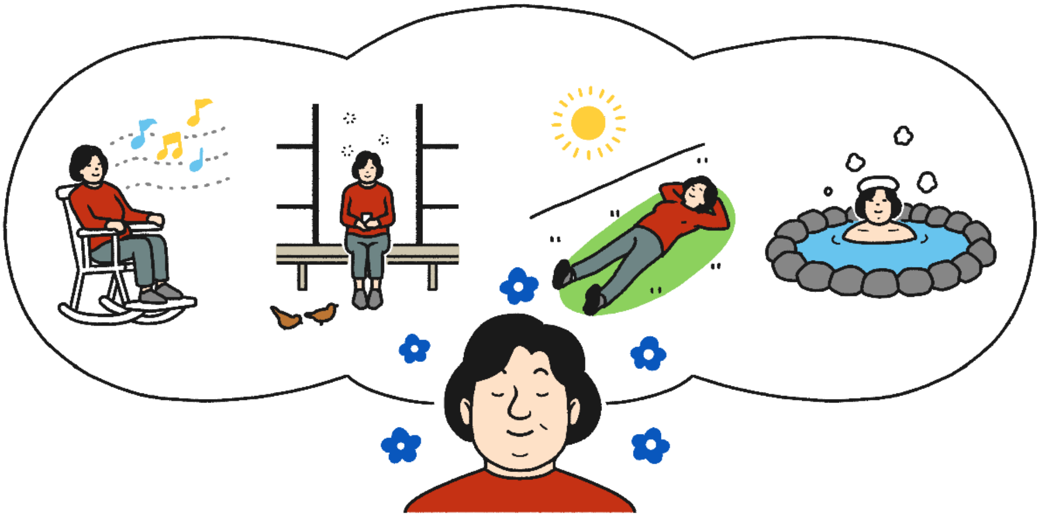

他のリラクセーションを行ってからの方がやりやすくなります。目を閉じて、誰かに語りかけてもらうとイメージしやすくなりますので、初めは治療者とやる方が良いでしょう。

イメージ法 つづき

自分に語りかけることでリラックスしていく方法もあります。
休憩時間や寝る前などにやってみるのが良いでしょう。
ポイントは、終わって活動するときには必ず消去動作をすることです。
これも初めは治療者と一緒に行うことをお勧めします。

1. 落ち着ける場所で力を抜きやすい楽な姿勢をとります

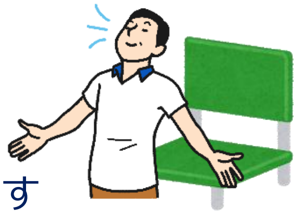

2. 目を閉じて大きく深呼吸します

3. 以下のことばを順にこころの中でつぶやきます

   「気持ちが　落ち着いている・・・」

   「利きうでが　重たい・・・」

   「利きうでが　あたたかい・・・」

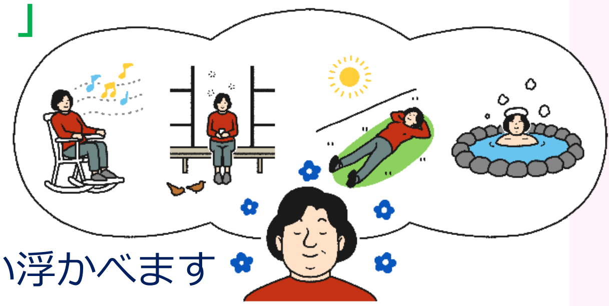

4. あたたかくて落ち着ける場所を思い浮かべます
   温泉でぽかぽか、ひなたぼっこでほっこりなど・・・

5. 「重たさ」「あたたかさ」の感覚がわかってきたら、
   利きうで⇒両うで⇒両あし　と注意を少しずつ全身に
   広げていき、感覚を味わいます

6. 消去動作をして終わります
   ①両手をゆっくり 2～3回グーパー
   ②両ひじをゆっくり曲げ伸ばし
   ③大きく背伸びをします

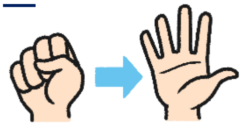

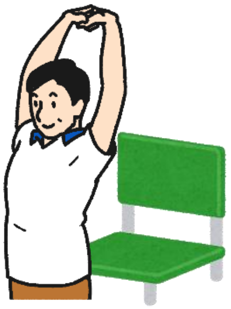

3. ストレス対処

日々のストレス対処は不安や緊張の予防につながりますし、また不安になったときに自分への癒しにもなります。以下のようなことを癒しにしている方が多いようです。ご自身にあった**癒しのレパートリー**を作っておけると良いかもしれません。

癒しのレパートリー 【活動】編

- □ 外食する
- □ ジュースを買って飲む
- □ ケーキを買って帰る
- □ 洋服を買う
- □ 漫画を買う
- □ DVDを借りる
- □ いつもと違う道を通る
- □ 散歩をする
- □ 軽い運動をする
- □ バッティングセンターに行く
- □ 明るい色の洋服を着る
- □ お気に入りの食器で食事をとる
- □ TVやYouTubeを見る
- □ スポーツを観戦する
- □ 人にグチをこぼしてみる
- □ 時々「ノー」と言ってみる
- □ 人の話をじっくりきく
- □ 苦手な人から距離をとる
- □ ぬいぐるみに文句を言う
- □ 「ありがとう」をたくさん言う
- □ 返信していないメールに返信する
- □ ふだん会っていない友人に電話やメールをする
- □ 日記にストレスを書きなぐる
- □ 古紙や古雑誌を破る
- □ カバンの中を整理して軽くする
- □ 机の上を整理する
- □ 靴をそろえる
- □ 絵を描く
- □ 鼻唄を歌う
- □ 観葉植物を買う
- □ 花を活ける

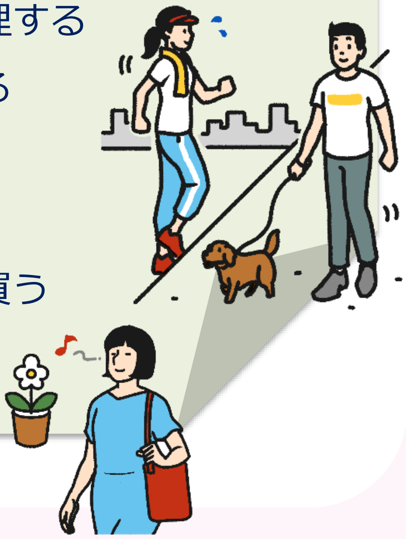

癒しのレパートリー 【休息】編

- □ ゆっくり入浴する（入浴剤を入れる）
- □ アロマやお香をたく
- □ 好きな音楽を聴いて気分転換する
- □ 深呼吸してみる
- □ 庭や芝生の上で寝ころがってみる
- □ 昼食後屋上で空をながめる
- □ 昼寝をする
- □ 時々休憩タイムをつくる
- □ 数日間休みをとる

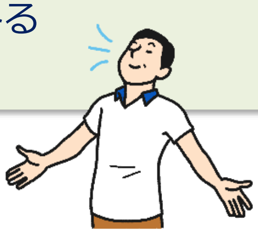

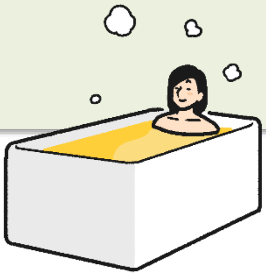

癒しのレパートリー 【五感】編

- □ 足つぼやマッサージを受ける
- □ ヨガ、ストレッチ
- □ ジョギング
- □ 水泳、水中ウォーキング
- □ ペットと遊ぶ
- □ ゲームをする
- □ インターネット
- □ 好きな食べ物や飲み物をとる
- □ 美術館、庭園
- □ 星空を眺める
- □ 昔の写真をみる
- □ 読書
- □ 旅行
- □ 料理
- □ 釣り
- □ 陶芸
- □ 囲碁、将棋
- □ 楽器の演奏
- □ ライブ
- □ 手芸、編み物
- □ スキー、スノボー
- □ アウトドア
- □ ガーデニング
- □ ドライブ
- □ ショッピング
- □ 動物園、水族館
- □ 香水を変える
- □ プラネタリウム
- □ カラオケ
- □ 映画鑑賞

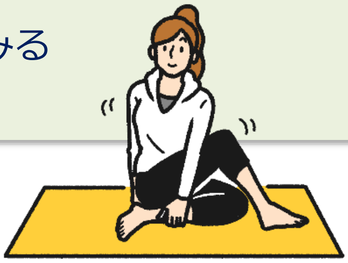

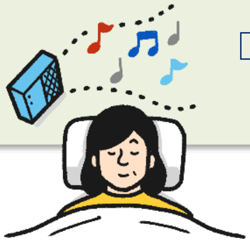

付録4

抗不安薬に関するよくある質問と回答

● やめられなくなるのでは？

　使い始めて短期間でやめられなくなることはありません。現在使用中で不安が改善している方は、中止に向けて少しずつ減らす方法（13ページ）があります。自己判断でやめると悪化することもあり、必ず医師と相談しながら減らします。

● 段々と効果が弱くなり量が増えるのでは？

　抗不安薬は、効き目が弱くなると感じることがありますが、それは、飲んでいる人や薬によって違いがあります。その場合は、薬の種類を変える、他の治療を検討するなどします。弱くなったと感じたら教えてください。

● 飲みすぎると死ぬのではないか心配です

　19ページで紹介した、現在処方される一般的な抗不安薬は、安全性の高い薬です。ただし、一度に大量に飲んだり、アルコールと一緒に飲んだりすると、呼吸抑制など重篤な副作用が現れ危険です。必ず医師の指示通りに使用します。

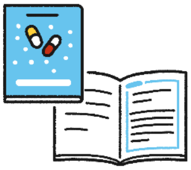

● 飲み合わせが心配です

　抗不安薬は抗うつ薬や生活習慣病治療薬などと相互作用があることが知られています。他の薬を飲み始める場合は、教えてください。病院に行くときはお薬手帳を持参し、医師に見せましょう。

● **認知症になると聞いたことがあります**

長期使用により認知症発症のリスクが高まるという報告と、そうではないという報告があり、結論は出ていません。すでに認知症と診断されている方、認知症のリスクが高い方は、長期使用は推奨されません。

● **胎児への影響はないのでしょうか？**

薬の胎児への影響は、薬の種類と妊娠の時期によって異なります。抗不安薬の胎児への影響は、まだ知見が積み重ねられている段階です。個別に医師に尋ねるとよいでしょう。

● **その他**（医師への質問をメモしておきましょう）

●

●

●

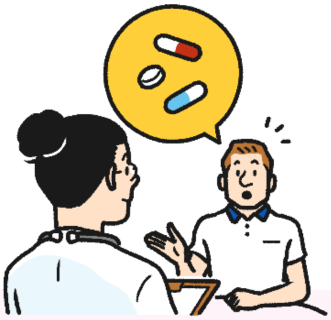

おわりに

●自分にあった治療法を選ぶために

治療の選択肢には、それぞれ長所と短所があります。この手引きは、それらをよく理解し、自分にとって重要なことを明らかにしながら医療者と話し合い、あなたに合った選択ができるよう作られています。

●手引きの開発プロセス

この手引きに掲載した情報は、不安症の治療を経験されたことのある方々の声や意見を反映させました。精神科の専門家のチェックも受けています。この手引きは厚生労働科学研究費補助金・障害者政策総合研究事業「平成29年度～30年度：向精神薬の処方実態の解明と適正処方を実践するための薬物療法ガイドラインに関する研究（H29-精神-一般-001）」および「平成31年/令和1年度～2年度：向精神薬の適切な継続･減量･中止等の精神科薬物療法の出口戦略の実践に資する研究（19GC1012）」により作成しました。特定の企業からの援助は受けていません。

●手引きの更新

この手引きは、必要に応じて見直しと更新をおこないます。

※ ここに掲載された情報は、医療者と話し合いながら対処法や治療法を決める際の手引きとなるものであり、医療者のアドバイスの代わりになるものではありません。

文献

• Takeshima M, Otsubo T, Funada D, et al.: Does cognitive behavioral therapy for anxiety disorders assist the discontinuation of benzodiazepines among patients with anxiety disorders? A systematic review and meta-analysis. Psychiatry Clin Neurosci. 2021, 75(4):119-127. doi: 10.1111/pcn.13195. (16ﾍﾟｰｼﾞ)

作成・向精神薬出口戦略マニュアル研究班・抗不安薬グループ

作成：2022年7月　　更新予定日：2024年7月

この手引きは「向精神薬の出口戦略ガイドライン」にもとづいて作成されています

©2022,向精神薬出口戦略マニュアル研究班
無断複写・無断転載はご遠慮ください
