# Supplementary material for: Development and acceptability of a decision aid for anxiety disorder considering discontinuation of benzodiazepine anxiolytic
Source: Front Psychiatry. 2023 May 12;14:1083568. doi: 10.3389/fpsyt.2023.1083568 (PMC10213963; doi:10.3389/fpsyt.2023.1083568)
Supplement: Supplementary file 3 [file Data_Sheet_3.PDF]

A decision aid for anxiety disorders considering discontinuation of anxiolytics
-A manual for healthcare providers-

【About this DA】

This decision aid (DA) can be used to help you and your patient decide on a treatment plan during shared decision making (SDM). SDM is a two-way decision-making process in which the patient and the healthcare provider decide together on the further treatment plan, which is a tool for making decisions together with the patient by sharing the advantages and disadvantages of each option, and discussing and confirming the patient's intentions, including preferences and values. Therefore, the DA is not intended to be handed over to the patient for him/her to decide independently. The DA is a piece of equipment that helps decide whether to continue or discontinue the use of anxiolytics, and when discontinuing the use of anxiolytics, whether to discontinue the use of anxiolytics only by tapering them or to reduce the use of anxiolytics by tapering them and adding cognitive-behavioral therapy (CBT), through discussion with the patient during the SDM process.

【Target of this DA】

The target of this DA is patients whose anxiety symptoms and daytime condition are improved by taking anxiolytics. For shared decision making using this DA, it is desirable that a sufficient period of time has passed since the improvement of symptoms to reduce the risk of relapse due to discontinuation.

【How to use the DA】

First, share with the patient how to use the DA. Emphasize that we will not come to a conclusion right away, but will make a decision together while using this tool. Explain that patients will be able to put a checkmark and write things down as they read, and tell patients that they can discuss their questions, impressions, and what they wrote down at the next visit.

Further treatment options

Step 1 (6-8)

In this section, the patient is asked to understand two options: "continuing anxiolytics" and "reducing anxiolytics." Regardless of which option is chosen, it is important to maintain remission (recovery) by continuing to do the "Daily activities to reduce anxiety," as indicated in Appendix 3.

For Option 1, "Continuing anxiolytics" (p. 7), review with the patient the pros and cons of current anxiolytics. Some patients taking GABA receptor anxiolytics (benzodiazepine and non-benzodiazepine anxiolytics) may want to switch to other types of anxiolytics (5-HT1A receptor agonists) because of their low side effects. The DA does not offer the option of switching from GABA receptor agonists to other types of anxiolytics, but some patients may consider such options. Nevertheless, the potential risks and benefits must be fully discussed.

There is currently no established method of switching from GABA receptor agonists to other types of anxiolytics. However, since the abrupt discontinuation of GABA receptor agonists can lead to relapse of anxiety symptoms and rebound anxiety, a gradual tapering approach should be used for the discontinuation of GABA receptor agonists in this case.

Step 2 (P9)

In this section, patients will be asked to review the pros and cons of each option.

Cautionary points

In some patients with anxiety disorders, discontinuation of anxiolytics can cause serious quality of life problems. In some cases, the patient may not be aware of this, and this point should be kept in mind. There may be an option to continue the use of the drug, keeping in mind the side effects while ensuring that the benefits outweigh the risks.[1] It is important to note that this DA does not aim to stop anxiolytics in all patients.

Step 3 (P10)

In this section, we discuss with the patient to clarify his or her values and preferences regarding each option. First, the patient is asked to rate the importance of the advantages and disadvantages of each of the options discussed in Step 2. Then, during the consultation, the patient is specifically asked to explain why he or she marked the number 0-5. In other words, this is a step to organize and share subjective information such as the patient's values and preferences together for shared decision-making, rather than simply automatically selecting the option with the highest score.

Step 4 (P11)

Ask the patient to proactively answer questions and concerns regarding the two options. Based on the information provided in steps 3 and 4, we discuss whether to continue or discontinue anxiolytics. If the decision is to discontinue the medication, proceed to the next section to discuss

how to discontinue the medication.

Further treatment options when reducing or stopping anxiolytics

Step 1 (p. 12-14)

Make sure the patient understands the two options: "Reducing or stopping using a gradual tapering method" or "Reducing or stopping using gradual tapering and CBT." Whichever option is chosen, it is important to continue with the "Daily activities to reduce anxiety" described in Appendix 3 to achieve successful withdrawal.

CBT for anxiety disorders

It is often administered once a week for a total of four to six sessions of approximately 60 minutes each. At present, CBT for panic disorder and social anxiety disorder is covered by insurance, but the number of facilities that can provide it varies by region.

Step 2 (p. 15)

Ask the patients to identify the pros and cons of each option.

Step 3 (P16)

Have the patients understand the effect of each option (what will happen as a result of choosing each option) using a pictogram.

About the pictogram

The pictogram is based on the results of a meta-analysis[2] of eight randomized controlled trials examining the effectiveness of CBT in the withdrawal of anxiolytics. The pictogram shows the percentage of patients estimated to have achieved abstinence at 3 months and at 1 year.

The percentage of patients estimated to have achieved abstinence after 3 months was 33% in the control group (tapering only) and 64% in the intervention group (tapering plus CBT), indicating a statistically significant difference between the two groups (risk ratio, 1.96; 95% confidence interval, 1.23-2.98; p=0.002).

The percentage of patients estimated to have achieved abstinence after 6-12 months was 31% in the control group (tapering only) and 67% in the intervention group (tapering plus CBT), indicating a statistically significant difference between the two groups (risk ratio, 2.16; 95% confidence interval, 1.41-3.32; p=0.0004).

Although only a few studies have examined the combined effects of undergoing CBT while reducing

or discontinuing anxiolytics, it should be noted that a previous meta-analysis[2] revealed significant short- and long-term effects.

Step 4 (P17)

In this step, we discuss with the patient to clarify his or her values and preferences regarding each option. First, the patient is asked to rate the importance of the advantages and disadvantages of each of the options discussed in Step 2. Then, during the consultation, the patient is specifically asked to explain why he or she marked the number 0-5. In other words, this is a step to organize and share subjective information such as the patient's values and preferences together for shared decision making, rather than simply automatically selecting the option with the highest score.

Step 5 (p. 18)

Ask the patient to proactively answer questions and address the concerns regarding the two options. Based on the information provided in steps 3 and 4, we make a shared decision on whether to withdraw using the gradual tapering method only or the combination of gradual tapering and CBT.

References

1. Hirschtritt ME, Olfson M, Kroenke K. Balancing the Risks and Benefits of Benzodiazepines. *JAMA*. 2021 Jan 26;325(4):347-348. doi: 10.1001/jama.2020.22106.
2. Takeshima M, Otsubo T, Funada D, Murakami M, Usami T, Maeda Y, Yamamoto T, Matsumoto T, Shimane T, Aoki Y, Otowa T, Tani M, Yamanaka G, Sakai Y, Murao T, Inada K, Yamada H, Kikuchi T, Sasaki T, Watanabe N, Mishima K, Takaesu Y. Does cognitive behavioral therapy for anxiety disorders assist the discontinuation of benzodiazepines among patients with anxiety disorders? A systematic review and meta-analysis. *Psychiatry Clin Neurosci*. 2021 Apr;75(4):119-127. doi: 10.1111/pcn.13195.

継続/減薬・中止：今後の治療法を一緒に決めるための手引き – 抗不安薬

― 医療者マニュアル ―

この手引きについて/手引きの使い方

【この手引き（DA）について】（P1）

この「継続/減薬・中止：一緒に決めるための手引き（Decision Aid :DA）–抗不安薬」は、患者と一緒に共同意思決定（shared decision making :SDM)の手法で治療法を決める際に活用できる資材である。DA は、各選択肢の利点・欠点を提示し、本人の好みや価値観を含めた意向を確認しながら話し合い、患者と一緒に意思決定を行うための補助資材である。したがって、**患者にこれを渡して単独で決めてもらうものではなく、あくまでも、医療者と患者での双方向性の話し合いを行うための補助資材である**。

本 DA は、抗不安薬の使用を継続するか、あるいは減量・中止するか、さらに減量・中止する場合は、漸減法のみで減量・中止するか、あるいは漸減法と不安症に対する認知行動療法も併用しながら減量・中止するかについて、SDM の手法で患者と一緒に話し合いながら決めるための資材である。

【この手引き（DA）の対象】（P2）

本 DA の対象は、抗不安薬を使用中で、不安症の症状が概ね改善していて、日中の心身の状態もよい患者である。すなわち、パニック発作、強い不安・恐怖などがなく、日中の QOL 障害が改善している必要がある。本 DA を用いて共同意思決定するにあたっては、寛解（回復）してから、休薬による再燃のリスクを低減させるのに十分な期間が経過していることが望ましい。

【この手引き（DA）の使い方】（P3）

はじめに本 DA の使い方について患者と共有する。すぐに結論を出すのではなく、この資材を活用しながら、一緒に決めていくことを強調する。以降のページでは、読み進めながら○をつけたり書き込んだりできるようになっていることを説明し、疑問や感想、書き込んだ内容について、次回話し合おうと伝える。

この先の治療の選択肢

【ステップ 1】（P6-8）

ここでは、「抗不安薬の使用を継続する」、「抗不安薬を減量・中止する」という 2 つの選択肢について患者に理解してもらう。いずれを選択した場合も付録 3（P23-30）で示されている「不安の軽減のためにできること」を継続することが寛解（回復）を維持する上で重要である。

選択肢 1「抗不安薬の使用を継続する」（P7）では、現在服用している抗不安薬の長所と短所を患者とともに確認する。ベンゾジアゼピン系抗不安薬（ベンゾジアゼピン受容体作動薬、GABA-A 受容体作動薬）を服用している患者の中には、副作用などの問題から別のタイプの抗不安薬（5-HT1A 受容体作動薬）への切り替えを希望する者もいるかもしれない。本 DA は、ベンゾジアゼピン系抗不安薬から新しいタイプの抗不安薬への切り替えという選択肢は設定していないが、患者によってはこのような選択肢も考慮される。その場合も予想されるリスクとベネフィットについては十分に話し合う必要がある。

なお、ベンゾジアゼピン系抗不安薬から別のタイプの抗不安薬への切り替えに関して現時点で確立した方法はない。

しかし、ベンゾジアゼピン系抗不安薬の突然の中止は不安症状の再燃や反跳性不安、離脱症状を引き起こす可能性があるため、この場合も減量・中止に当たっては漸減法を用いるべきである。

【ステップ 2】(P9)

ここでは、各選択肢の長所・短所を確認してもらう。

注意点

抗不安薬(GABA-A 受容体作動薬)にもリスクとベネフィットがあり[1]、特に不安症患者の一部では、抗不安薬を継続することで QOL を維持している人がいるのも事実である。そのような患者は抗不安薬の中断によって深刻な QOL 障害が生じることがある。本人がこれを自覚していない場合もあり、この点については留意する必要がある。ベネフィットがリスクを上回っていることを確認しつつ、副作用に留意し使用を継続するという選択肢もあり得る[1]。本 DA が全ての患者の抗不安薬の中止を目指すことを目的としていない点には留意が必要である。

【ステップ 3】(P10)

ステップ 2 で取り上げた各選択肢の長所・短所について、重要度を評価してもらう。その他、患者が自由記載した項目についても詳細をきき、共同意思決定の材料にする。

【ステップ 4】(P11)

2 つの選択肢に関する疑問点、懸念事項を積極的に記入してもらう。ステップ 3、4 の記載内容に基づき、抗不安薬を継続するか、減量・中止するか話し合う。減量・中止する方針となった場合は、次のセクションに進み休薬の方法について話し合う。

『抗不安薬を減量・中止する』場合のこの先の治療の選択肢

【ステップ 1】(P12-14)

「漸減法を用いて減量・中止する」、「漸減法と認知行動療法を併用して減量・中止する」という 2 つの選択肢の内容を理解してもらう。いずれを選択した場合も付録 3(P23-30)で示されている「不安の軽減のためにできること」を継続することが、休薬を成功させる上で重要である。

不安症の認知行動療法について

現時点で不安症(パニック症、社交不安症など)の認知行動療法は保険収載されているが、実施できる施設は限られている。アクセスの問題もあり、実施できる地域の医療機関のリストが作成できないことが課題である。

【ステップ 2】(P15)

各選択肢の長所・短所を確認してもらう。

【ステップ 3】(P16)

ピクトグラムを用いて各選択肢の効果(各選択肢を選んだ結果どうなるか)について理解してもらう。

ピクトグラムについて

本ピクトグラムは、抗不安薬の休薬における認知行動療法併用の有効性を検討した 3 つの無作為化比較対照試験のメタ解析[2]の結果に基づき作成されている。ピクトグラムは、3ヶ月後および 6～12ヶ月後に中止が達成されていると推定される患者の割合を選択肢毎に示している。

3 ヶ月後に中止が達成されていると推定される患者の割合は、対照群（漸減法のみ）33%、介入群（漸減法+認知行動療法）64%であり、両群の間に統計学的に有意な差が認められた（リスク比：1.96、95%信頼区間：1.29－2.98、p=0.002）。

6～12 ヵ月後に中止が達成されていると推定される患者の割合については、対照群 31%、介入群 67%であり、両群の間に統計学的に有意な差が認められた（リスク比：2.16、95%信頼区間：1.41－3.32、p=0.0004）。

抗不安薬の減量・中止における認知行動療法の併用効果を検討した研究はまだ少数であるが、本メタ解析[2]では、短期および長期効果について統計学的な有意性が示された点は注目される。

【ステップ 4】（P17）

ステップ 2 で取り上げた各選択肢の長所・短所について、重要度を評価してもらう。その他、患者が自由記載した項目についても詳細をきき、共同意思決定の材料にする。

【ステップ 5】（P18）

2 つの選択肢に関する疑問点、懸念事項を積極的に記入してもらう。ステップ 3、4 の記載内容に基づき、漸減法を用いて減量・中止するか、漸減法と認知行動療法を併用して減量・中止するかを共同意思決定する。

文献)

3. Hirschtritt ME, Olfson M, Kroenke K. Balancing the Risks and Benefits of Benzodiazepines. *JAMA*. 2021 Jan 26;325(4):347-348. doi: 10.1001/jama.2020.22106.

4. Takeshima M, Otsubo T, Funada D, Murakami M, Usami T, Maeda Y, Yamamoto T, Matsumoto T, Shimane T, Aoki Y, Otowa T, Tani M, Yamanaka G, Sakai Y, Murao T, Inada K, Yamada H, Kikuchi T, Sasaki T, Watanabe N, Mishima K, Takaesu Y. Does cognitive behavioral therapy for anxiety disorders assist the discontinuation of benzodiazepines among patients with anxiety disorders? A systematic review and meta-analysis. *Psychiatry Clin Neurosci*. 2021 Apr;75(4):119-127. doi: 10.1111/pcn.13195.
